# Wild type human TDP-43 potentiates ALS-linked mutant TDP-43 driven progressive motor and cortical neuron degeneration with pathological features of ALS

**DOI:** 10.1186/s40478-015-0212-4

**Published:** 2015-06-25

**Authors:** Jacqueline C Mitchell, Remy Constable, Eva So, Caroline Vance, Emma Scotter, Leanne Glover, Tibor Hortobagyi, Eveline S. Arnold, Shuo-Chien Ling, Melissa McAlonis, Sandrine Da Cruz, Magda Polymenidou, Lino Tessarolo, Don W Cleveland, Christopher E Shaw

**Affiliations:** King’s Centre for Neurodegeneration Research, Kings College London, Department of Basic and Clinical Neurosciences, Institute of Psychiatry, Psychology and Neuroscience, London, SE5 8AF UK; Centre for Ultrastructural Imaging, Kings College London, London, SE1 1UL UK; Ludwig Institute for Cancer Research and Department of Molecular Medicine, University of California at San Diego, La Jolla, CA 92093 USA; Mouse Cancer Genetics Program, National Cancer Institute – Frederick, Frederick, MD 21702 USA; Present address: Neurogenetics Branch, NINDS, National Institutes of Health, Bethesda, MD 20892 USA; Present address: Institute of Molecular Life Sciences, University of Zurich, Zurich, CH-8057 Switzerland

**Keywords:** TDP-43, Amyotrophic lateral sclerosis, Mouse

## Abstract

**Introduction:**

Amyotrophic lateral sclerosis (ALS) is a relentlessly progressive neurodegenerative disorder, and cytoplasmic inclusions containing transactive response (TAR) DNA binding protein (TDP-43) are present in ~90 % of cases. Here we report detailed pathology in human TDP-43 transgenic mice that recapitulate key features of TDP-43-linked ALS.

**Results:**

Expression of human wild-type TDP-43 (TDP-43^WT^) caused no clinical or pathological phenotype, while expression of Q331K mutant (TDP-43^Q331K^) resulted in a non-lethal age-dependent motor phenotype, accompanied by cytoplasmic TDP-43 aggregation, mild neuronal loss, with astroglial and microglial activation in the motor cortex and spinal cord at 24 months. However, co-expression of WT and Q331K mutant (TDP-43^WTxQ331K^) resulted in an extremely aggressive motor phenotype with tremor from 3 weeks and progressive hind-limb paralysis necessitating euthanasia by 8–10 weeks of age. Neuronal loss and reactive gliosis was observed in the spinal cord and layer V region of the cortex, with TDP-43, ubiquitin and p62 cytoplasmic inclusions and an increase in insoluble TDP-43. Nuclear clearance of TDP-43 was not observed in TDP-43^Q331K^ mice but was seen in 65 % of aggregate containing spinal cord motor neurons in TDP-43^WTxQ331K^ mice.

**Conclusions:**

We hypothesise that cytoplasmic TDP-43^Q331K^ aggregates facilitate the recruitment of WT protein in compound animals, which dramatically accelerates neurodegeneration and disease progression. The exploration of disease mechanisms in slow and rapid disease models of TDP-43 proteinopathy will help elucidate novel drug targets and provide a more informative platform for preclinical trials.

**Electronic supplementary material:**

The online version of this article (doi:10.1186/s40478-015-0212-4) contains supplementary material, which is available to authorized users.

## Introduction

Amyotrophic lateral sclerosis (ALS) is a relentlessly progressive neurodegenerative disorders characterised by the degeneration of motor neurons in the motor cortex and spinal cord, progressive paralysis and ultimately death due to respiratory failure.

Transactivating response region (TAR) DNA binding protein (TDP-43) has been identified as a major component of neuronal and glial inclusions found in ~95 % of sporadic ALS patients [1, 2]. TDP-43 is predominantly a nuclear protein involved in regulating RNA transcription, splicing, trafficking and microRNA biogenesis [3]. Cortical and motor neurons containing cytoplasmic TDP-43 aggregates often appear to have little or no nuclear TDP-43 [4, 5], implicating a loss of nuclear TDP-43 function in disease pathogenesis, however cytoplasmic aggregation is also toxic and may play a key pathogenic role.

The identification of mutations in the glycine-rich C terminal region of TDP-43 that enhance protein cleavage, aggregation and neurotoxicity confirmed a pathologenic role for TDP-43 mis-accumulation in ALS and FTLD [6, 7, 8]. TDP-43 positive inclusions have also been identified in patients with pathogenic mutations in the genes encoding progranulin (*PGRN* [9])*,* valosin containing protein (*VCP* [10]) and Ubiquilin 2 (*UBQLN2* [11]) and the G4C2 hexanucleotide expansion mutation identified in C9ORF72 [12, 13]. The identification of mutations in a structurally and functionally similar nucleic acid binding protein, fused in sarcoma (*FUS*) [14, 15] supports the hypothesis that defective RNA processing or aberrant assembly of these aggregation prone proteins [16, 17] are mechanistic contributors in the pathogenesis of ALS.

TDP-43 is a 414 amino acid protein, consisting of two RNA recognition motifs (RRM1 and RRM2) [18, 19], a nuclear import and export signal [20], and a glycine rich region that has been implicated in its protein-protein interactions [16, 17], including those involved in its regulation of RNA splicing [21, 22]. It has been shown to bind to over 6000 pre-mRNAs and over 2000 non-coding RNAs within the central nervous system, affecting the levels of around 600 mRNAs, and regulating the splicing patterns of another 950 [23, 24]. TDP-43 has also been demonstrated to autoregulate its own RNA level [23, 25] at least in part by stimulating the excision of an intron in its 3’ untranslated region (UTR) which initiates its degradation by nonsense mediated RNA decay [23].

A number of rodent models have been generated to assess the effect of mutation and TDP-43 expression levels *in vivo*. Disruption or constitutive knockout of the Tardp gene in mice is embryonic lethal [26, 27, 28], while post-natal knockout results in metabolic defects, leading to rapid death [29]. In contrast, heterozygous knockdown results only in a mild impairment in grip strength, with no overt evidence of neurodegeneration [27], while motor neuron specific knockdown results in age dependent progressive motor dysfunction and motor neuron loss [30, 31]. Studies investigating the effect of overexpression of human wild-type (WT) or disease mutant TDP-43 have demonstrated varying levels of lethality, motor phenotype and pathology [32, 33, 34, 35, 36, 37, 38, 39], with some evidence of a dose-dependent effect [32] that may be enhanced by the presence of a pathogenic mutation [38, 39]. Some models are also confounded by the presence of phenotypes not identified in patients, such as the existence of a severe gut motility defect [40, 41]. In many cases the pathology observed in rodent models does not convincingly recapitulate many aspects of the human disease.

Here we report the physical, pathological and biochemical characterisation of mice transgenic for human wild-type (WT) and ALS-linked Q331K mutant TDP-43. Mice overexpressing human TDP-43^WT^ develop no overt physical or pathological phenotype, while those overexpressing mutant human TDP-43^Q331K^ develop an age-dependent mild motor and pathological phenotype. Mice co-expressing TDP-43^WT^ and TDP-43^Q331K^ mutant TDP-43 develop marked tremor at 3 weeks and rapidly progressive paralysis requiring euthanasia by 8–10 weeks. Pathologically, TDP-43^WTxQ331K^ mice demonstrate major neuronal loss, robust astroglial reactivity, increased cytoplasmic TDP-43 accumulation and detergent resistant TDP-43. We propose that mislocalisation and aggregation of the mutant protein seeds the rapid recruitment of wild-type TDP-43 that greatly accelerates the disease processes.

## Materials and methods

### Ethics statement

All experiments were performed under the terms of the UK Animals (Scientific Procedures) Act 1986, and were approved by the Kings College, London ethics review panel.

### Generation of transgenic animals

**Fig. 1:**
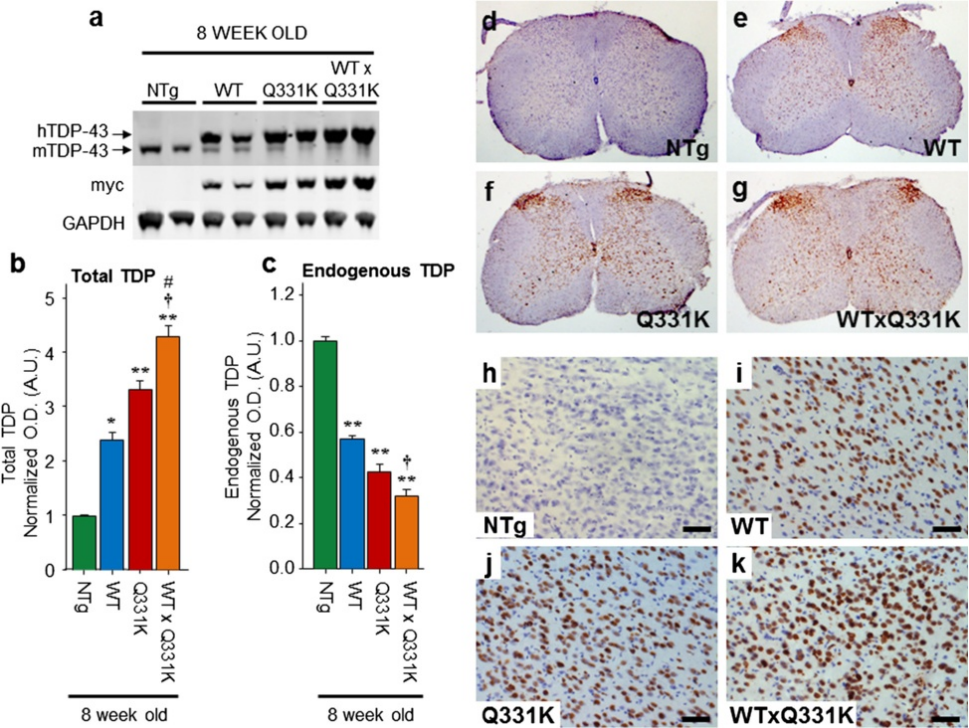
Expression of TDP-43^WT^ and TDP-43^Q331K^ in mice decreases endogenous TDP-43 expression. **a** Western blotting of brain lysate from 8 week old non-transgenic (NTg), TDP-43^WT,^ TDP-43^Q331K^, and TDP-43^WTxQ331K^ mice using an anti-TDP antibody showed a slight shift to a higher molecular weight due to the presence of the myc tag. There was an increase in total TDP43 expression in all transgenic animals, with higher expression in TDP-43^Q331K^ mice than TDP-43^WT^ animals, and TDP-43^WTxQ331K^ animals showing evidence of cumulative TDP43 expression (**b**). This increase was accompanied by a concomitant decrease in endogenous TDP43 expression (**c**). **d–k** Immunohistochemistry with anti-myc antibody showed expression of the TDP-43 protein throughout the spinal cord (**d–g**) and in the cortex (h–k; scale bar: 50 μm) in TDP^WT^ (**e, i**), TDP^Q331K^ (**f, j**) and TDP-43^WTxQ331K^ (**g, k**) mice, which was absent in NTg animals (**d, h**) . (All graphs are mean ± SEM; * *p* < 0.05; ** *p* < 0.001 vs. NTg; †† *p* < 0.001 vs. TDP^WT^, ## *p* < 0.001 vs. TDP^Q331K^)

Construction of transgenic mice expressing TDP-43^WT^ (line 96) or TDP-43^Q331K^ (line 31) has been previously described (see Additional file 1: Figure S1 in reference [36]). Briefly, cDNAs containing N-terminal myc-tagged full length wild-type or Q331K mutant TDP-43 were amplified by PCR to insert flanking SalI digestion sites. The resulting products were digested by SalI and cloned into the XhoI insertion site of the MoPrP. XhoI vector (ATCC #JHU-2). The resultant MoPrP. XhoI-mychuTDP-43 construct was then digested upstream of the minimal PrP promoter and downstream of the final PrP exon 3 using BamHI and NotI and subcloned into a shuttle vector containing loxP flanking sites (see Fig. 1a in reference [36]). The final construct was then linearized using XhoI, injected into the pro-nuclei of fertilized C57Bl6/C3H hybrid eggs and implanted into pseudopregnant female mice. After obtaining multiple founder mice, lines displaying comparable levels of mutant or wild-type transgene accumulation were selected and backcrossed to C57Bl6 to establish the lines detailed in this paper. The mice used for analysis were backcrossed to C57Bl6 for a minimum of four generations. All mice were maintained on a C57B6/J background, and compound hemizygous animals were generated by crossing hemizygous TDP-43^WT^ animals with TDP-43^Q331K^ mutant expressing animals (TDP-43^WTxQ331K^). Mice from the single hemizygous lines were identified using PCR with primers 5’- GGATGAGCTGCGGGAGTTCT and 3’- GTCAACCCCATACTACCCGT. Animals from the compound hemizygous lines were identified with direct sequencing using the primers 5’- ATGACTGAGGATGAGCTGCG and 3’- GGATGCTGATCCCCAACCAA.

### Evaluation of motor function and health

From 3 weeks of age, mice were regularly weighed and general health status was recorded. Animals showing signs of hind-limb paralysis were monitored daily, and disease end stage and death was defined as the time when animals could no longer obtain food or water, or had lost 25 % of their body weight, at which point they were euthanized.

Motor strength and coordination were evaluated on the rotarod (Columbus Instruments) at multiple ages (5 weeks, 3, 6, 12, 18 24 months), using a 5 min accelerating protocol starting at 2 rpm, and rising to 30 rpm throughout the 5 min testing period. Mice were tested on multiple occasions, and all animals received an initial training session of 2 min at 2 rpm to acclimatise them to the equipment.

Data from 5 week old TDP-43^WTxQ331K^ mice and their littermates were assessed statistically by one-way analysis of variance (ANOVA) followed by the Tukey test. All other data were assessed statistically by two-way ANOVA followed by the post-hoc Holm-Sidak test.

### Histology and immunohistochemistry

Eight week old, end stage TDP-43^WTxQ331K^ mice, and 24 month old single hemizygous mice and their respective age-matched littermates were anaesthetised and transcardially perfused with PBS followed by 4 % paraformaldehyde (PFA) in phosphate buffer. Brain, spinal cord and gastrocnemius muscles were postfixed in 4 % PFA in 15 % sucrose for 5 h, cryoprotected in 30 % sucrose for 24 h and cut into 30 μm (brain and cord) or 40 μm (muscle) sections on a cryostat.

For immunohistochemistry, the following antibodies were used: rabbit anti-TDP-43 (1:500, Proteintech), rabbit anti-mouse TDP-43 (0.1 μg/ml, a gift from Prof. Virginia Lee (Igaz et al. 2011)), rabbit anti-ubiquitin (1:1000, DAKO), rat anti-myc (1:1000, Serotec), rabbit anti-GFAP (1:4000, DAKO), mouse anti-CD68 (1:2000, Serotec), rabbit anti-p62 (sequestosome 1) (1:10,000; Abcam). For bright field imaging, sections were washed and incubated with the appropriate biotinylated secondary antibody (1:1000, Vector), and then with an ABC kit (Vector). Sections were imaged using a Zeiss light microscope and Axiovision software. For fluorescence imaging, sections were incubated with rat anti-myc (1:200) and either rabbit anti-ubiquitin (1:200) or rabbit anti-p62 (1:5000), sections were washed and incubated with goat anti-rat Alexa Fluor 488 and goat anti-rabbit AlexaFluor 568 (Invitrogen), and imaged using a Leica confocal microscope and LAS-AF software.

For motor neuron counts, perfused lumbar spinal cords from 3 to 4 animals per genotype were serially sectioned, and every 6th section (30 μm) was analysed. Sections were mounted, dried, incubated overnight in 1:1 ethanol/chloroform to de-fat the sections, stained for 10 min in warm 0.1 % cresyl violet, dehydrated and coverslipped. To compare the number of motor neurons, large neurons greater than 30 μm in diameter (as assessed using the integrated morphometry analysis package in Metamorph 7.7, Molecular Devices, Wokingham, UK) in the anterior horn of the lumbar spinal cord were counted in 15 sections. Data were assessed statistically by one-way ANOVA, followed by the post-hoc Tukey test. For cortical neuron counts, perfused brains from 3 to 4 animals per genotype were serially sectioned, and every 12th section (30 μm) through the motor cortex was assessed. Sections were stained as for the lumbar spinal cord. To compare the number of neurons, cells greater than 5 μm in diameter (as assessed using the nuclear count analysis package in Metamorph) were counted in four images per section. Data were assessed statistically by one-way ANOVA, followed by the post-hoc Tukey test.

For muscle histology, gastrocnemius muscles (2–4 animals per age and genotype) were dissected fresh, immediately frozen in isopentane cooled in dry ice, and cryostat sections were cut onto slides and stained with haematoxylin and eosin. For the detection of neuromuscular junctions (NMJs), perfused gastrocnemius muscle (2–3 animals per age and genotype) was incubated in Alexa Fluor 555 α**-**bungarotoxin (1:500, Life Technologies) and rabbit anti-Synaptophysin 1 (1:500, Synaptic Systems). Sections were washed and incubated in donkey anti-rabbit IgG DyLight 488 secondary antibody (1:500, Thermo Scientific) and imaged on a Leica confocal microscope. To assess NMJ area, the total area stained by bungarotoxin was assessed in ImageJ, in 30–40 NMJs per animal. To calculate the number of intact NMJs, 70–100 were assessed per animal, and were considered intact if they demonstrated full colocalisation of bungarotoxin and synaptophysin staining. Data were assessed statistically by one-way ANOVA, followed by the post-hoc Tukey test.

### Nerve root axon count

7 week old, end stage TDP-43^WTxQ331K^ mice (n = 3–4 per genotype), and 24 month old single hemizygous mice (n = 3–6 per genotype) and their respective age-matched littermates were anaesthetised and transcardially perfused with PBS followed by a mixture of 2 % PFA and 2.5 % glutaraldehyde in 0.1 M cacodylate buffer. The L5 ventral roots were removed and post fixed in the same fixative at 4 °C overnight. The roots were then further fixed in 1 % osmium tetroxide in0.1 M cacodylate buffer for 4 h. Fixed tissue was then dehydrated in ethanol and embedded in Epox 812/Araldite 502 (TAAB). semi-thin sections (0.65 μm) were cut using an ultramicrotome (Reichart-Jung Ultracut-E) and collected onto glass slides. The sections were stained with 1 % toluidine blue for 15 s before mounting for viewing and examined under a light microscope. Axon measurements were made using the integrated morphometry package on Metamorph 7.7 (Molecular Devices, Wokingham, UK) and α-motor axons, defined as those with a diameter greater than 3.5 μm, were counted. Data was analysed statistically by way of ANOVA followed by the post-hoc Tukey test.

### Immunoblotting

To assess expression levels of full length TDP-43, the 25 and 35 kDa TDP-43 fragments, and phospho-TDP-43, whole brains of 3–4 end stage hTDP^WTxQ331K^ and 3–4 age-matched hTDP^WT^, hTDP^Q331K^ and non-transgenic animals, were lysed in low salt buffer (10 mM Tris, 5 mM EDTA, 10 % sucrose) with protease inhibitors (Roche Diagnostics, UK). Total TDP-43 levels were also assessed in four 24 month old hTDP^WT^, hTDP^Q331K^ and non-transgenic animals.

For cytoplasmic and nuclear fractionation, four brain samples for each age and genotype were prepared as described earlier [42]. Briefly, snap-frozen tissue was weighed and homogenised in buffer containing 10 mM Hepes, 10 mM NaCl, 1 mM KH_2_PO_4_, 5 mM NaHCO_3_, 5 mM EDTA, 1 mM CaCl_2_, 0.5 mM MgCl_2_ and protease inhibitors (10x vol/weight). After 10 min on ice, 2.5 M sucrose (0.5x vol/weight) was added. Tissue was homogenized and centrifuged at 6300 g for 10 min. The supernatant was collected as the cytoplasmic fraction. The pellet was washed four times in TSE buffer (10 mM Tris, 300 mM sucrose, 1 mM EDTA, 0.1 % IGEPAL (Sigma) and protease inhibitors 10x vol/weight), homogenized and centrifuged at 4000 *x* g for 5 min. Finally the pellet was resuspended in RIPA buffer with 2 % SDS (5x vol/weight) as the nuclear fraction.

For insolubility assessment, four brain samples for each age and genotype underwent sequential extraction in buffers of increasing stringency, based on a modified protocol previously described [43]. Briefly, snap-frozen tissue (500 mg/ml w/v) was extracted by repeated homogensiation and cetrifugation steps (120,000 g, 30 min 4 °C) in high salt buffer (50 mM Tris–HCl, 750 mM NaCl, 10 mM NaF, 5 mM EDTA, pH7.4), 1 % Triton X-100 in high salt buffer, RIPA buffer (50 mM Tris–HCl, 150 mM NaCl, 5 mM EDTA, 1 % NP-40 substitute, 0.5 % sodium deoxycholate, 0.1 % sodium dodecyl sulphate) and urea buffer (30 mM Tris HCl pH 8.5, 7 M Urea, 2 M Thiourea, 4%CHAPS). To prevent carry over, each extraction step was performed twice. Supernatants from the first extraction steps were analysed, while supernatants from the wash steps were discarded. Protease inhibitors were added to all buffers excluding the urea buffer prior to use.

Protein samples were then separated by SDS/PAGE using 10 % polyacrylamide gels, and transferred to nitrocellulose membranes. Total TDP-43 and the 25 and 35 kDa fragments were recognised by a rabbit polyclonal antibody to TDP-43 (1:1000, Proteintech), and exogenous myc tagged human TDP-43 was recognised by a mouse monoclonal antibody to the myc tag (1:1000, Cell Signalling). Phospho-TDP-43 was recognised using a rabbit anti-phospho-Ser409/410-TDP-43 (1:1000, CosmoBio), and in these analyses, total TDP was recognised using a rat monoclonal antibody to TDP-43 (1:1000, BioLegend) . Fluorescent secondary antibodies conjugated to Dylight 680 nm or 800 nm (Thermo Scientific) were used to detect protein levels, and results were visualised using the Odyssey Imager (Licor). Data were normalised to GAPDH (1:5000, Sigma), actin (1:20,000, Abcam) or Lamin B1 (1:2000, Abcam). Quantitation of immunoblots was done using Image J software, and data were analysed statistically by way of ANOVA followed by the post-hoc Tukey test.

## Results

### Overexpression of human wild-type and Q331K mutant TDP-43 causes progressive paralysis and death in mice

Mice expressing human WT (TDP-43^WT^) or Q331K mutant (TDP-43^Q331K^) TDP-43 were interbred to generate TDP-43^WTxQ331K^ animals expressing both the WT and mutant protein. Initial breeding approximated a Mendelian distribution of genotypes on weaning. Because 3 week old TDP-43^WTxQ331K^ animals struggled to compete for food against their single transgenic littermates we added wet mash food to their home cage prior to weaning which ensured survival.

Immunoblots of brain lysates from TDP-43 transgenic mice showed that the exogenous human TDP-43 protein had a slightly higher molecular weight than the endogenous mouse TDP-43, due to the presence of the myc tag (Fig. 1a). Quantification of expression indicated that total TDP-43 expression detected in the brains of young TDP-43^WTxQ331K^ mice was 3.3 fold higher than non-transgenic animals (Figs. 1a–b), while expression in young TDP-43^WT^ and TDP-43^Q331K^ single hemizygous animals was 1.4 and 2.2 fold higher respectively. Levels of transgene encoded mouse TDP-43 were maintained during aging (Additional file 1: Figure S1). The increase in total TDP-43 observed in transgenic animals was associated with a proportionate downregulation of endogenous murine TDP-43 (Fig. 1c, Additional file 1: Figures S1 and S2), which is consistent with previous reports of autoregulation of TDP-43 mRNA levels [23, 25] and the absence of a key autoregulatory sequence [23] on our transgene encoded mRNA. Immunohistochemistry using a myc-tag antibody to selectively visualise only the human TDP-43 protein revealed expression throughout the spinal cord (Figs. 1d–g), and in the cortex (Fig. 1h–k) in all transgenic animals.

**Fig. 2:**
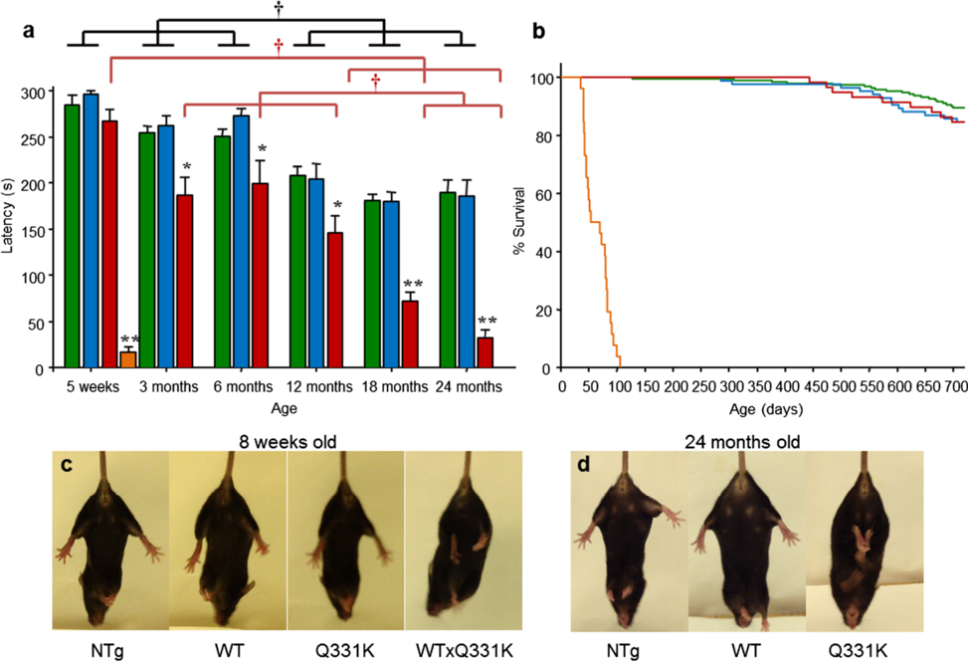
Co-expression of TDP-43^WT^ and TDP-43^Q331K^ in mice results in motor dysfunction and premature death. **a** TDP-43^WTxQ331K^ (orange triangle symbol) mice display a significant impairment in performance on the rotarod at 5 weeks of age, compared to NTg (green triangle symbol), TDP-43^WT^ (blue triangle symbol) and TDP-43^Q331K^ (red triangle symbol) age matched animals. Aging resulted in a significant impairment in motor function in all animals, regardless of genotype, but this impairment was significantly enhanced in TDP-43^Q331K^ mice compared to their NTg and TDP^WT^ littermates (mean ± SEM; * *p* < 0.05; ** *p* < 0.001 vs all other age matched genotypes; † *p* < 0.05). **b** Survival curve showing an average survival of 64 ± 6 days for TDP-43^WTxQ331K^ mice, while TDP-43^Q331K^ and TDP-43^WT^ survival does not significantly differ from NTg mice. **c** Abnormal hind limb splay in 8 week old TDP-43^WTxQ331K^ mouse. **d** Abnormal hind limb splay in a 24 month old TDP-43^Q331K^ mouse

Motor function was assessed at multiple age points using the accelerating rotarod paradigm. Animals were born at Mendelian ratios, and were indistinguishable from their non-transgenic littermates until 3 weeks of age when the TDP-43^WTxQ331K^ animals began to develop tremor. From 3 weeks of age, TDP-43^WTxQ331K^ mice displayed a rapid decline in their motor function, with a significant reduction in performance on the accelerating rotarod at 5 weeks of age compared with non-transgenic, TDP-43^WT^ and TDP-43^Q331K^ littermates (Fig. 2a). TDP-43^WTxQ331K^ animals developed progressive hind limb paralysis with a severely stilted gait, an inability to raise their pelvis off the ground (Additional file 2: Movie S1) and a failure to splay hind limbs normally when lifted by their tail (Fig. 2c). Following the onset of severe hind limb paralysis at 5–6 weeks, disease progressed rapidly in TDP-43^WTxQ331K^ animals, necessitating euthanasia by 8–10 weeks because they were unable to obtain food or water, or had lost 25 % of their total body weight. The average survival time for TDP-43^WTxQ331K^ mice was 64 ± 4 days (Fig. 2b). In contrast TDP-43^WT^ animals displayed no significant motor dysfunction or signs of ill health out to 24 months. TDP-43^Q331K^ animals displayed a mild but progressive motor dysfunction as assessed by the rotarod (Fig. 2a), accompanied by abnormal hind limb splay (Fig. 2d) and tremor but no evidence of early lethality (Fig. 2b). Previous studies have demonstrated intestinal blockage and/or megacolon as a major phenotype in prion-promoter driven TDP-43 mouse overexpression models [40, 41, 44] however, the TDP-43^WTxQ331K^ mice displayed normal bowel movements throughout their lives, and physical examination of the intestines immediately following euthanasia did not detect any evidence of intestinal blockage or megacolon. In addition, the progressive decline in motor function observed in these mice resulted in increasing difficulty in accessing food and water. The provision of wet mash at floor level was sufficient to maintain them at a consistent weight until the last few days prior to euthanasia, when they increasingly struggled to access food, and began to lose weight. The progressive phenotype observed in these animals was more characteristic of the G93A SOD1 mouse model [45] than the TDP-43 A315T mice which display significant gut motility issues [40, 41, 44, 37].

### ALS-like spinal cord pathology in TDP-43^Q331K^ mice is potentiated by co-expression with wild type TDP-43

**Fig. 3:**
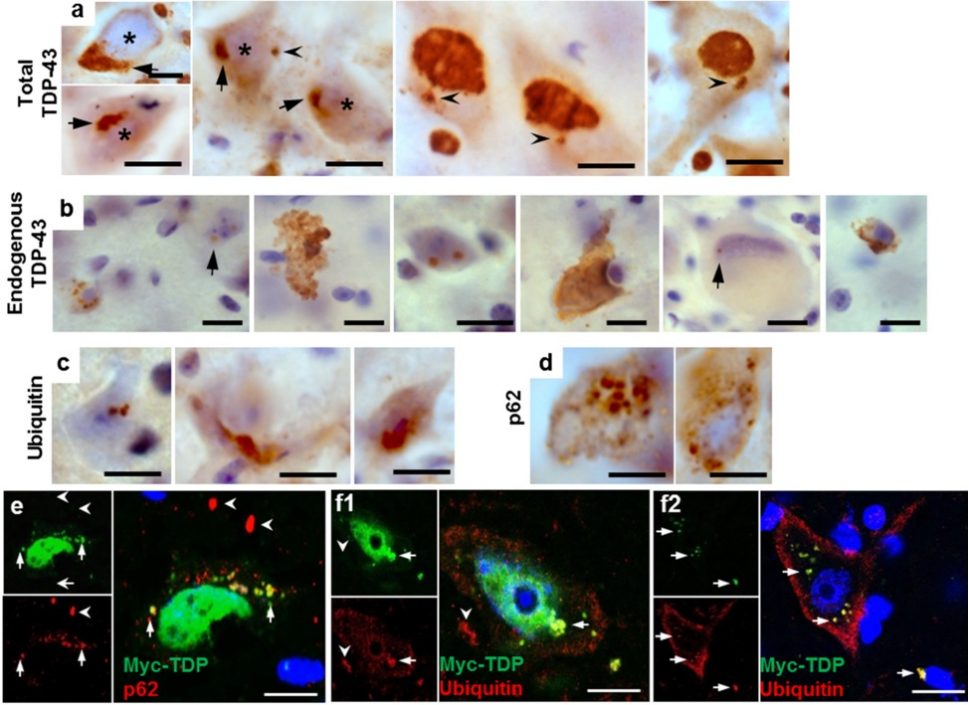
Co-expression of TDP-43^WT^ and TDP-43^Q331K^ in mice results in ubiquitin and p62 positive TDP-43 inclusions in the anterior horn of the lumbar spinal cord. **a** Large perinuclear TDP-43 aggregates (arrows) and small cytoplasmic TDP-43 inclusions (arrowheads) were present in multiple cells from the anterior horn of the lumbar spinal cord of TDP-43^WTxQ331K^ animals. In many cases, these aggregates are accompanied by evidence of nuclear clearing of TDP-43 (asterisks) There is also evidence of large and small (arrows) cytoplasmic and nuclear inclusions of endogenous mouse TDP-43 (**b**), accompanied by ubiquitin (**c**) and p62 (**d**) aggregation in TDP-43^WTzQ331K^ mice (scale bar: 10 μm). **e–f** Fluorescent co-labelling of myc-TDP and p62 (**e**) or ubiquitin (**f**) in the anterior horn of TDP-43^WTxQ331K^ animals shows aggregates that are positive for both myc-TDP and p62/ubiquitin (arrows), together with myc-TDP negative, p62 or ubiquitin inclusions (arrowheads). TDP-43 and ubiquitin aggregates can be seen both in the presence (f1) and absence (f2) of nuclear TDP-43 (scale bar : 10 μm)

In the spinal cord of 8 week old TDP-43^WTxQ331K^ mice, TDP-43 staining showed frequent globular cytoplasmic inclusions similar to globular punctate inclusions seen in human TDP-ALS patients [1, 2] (Fig. 3a), which were absent in age matched TDP-43^WT^ and TDP-43^Q331K^ single transgenic and non-transgenic animals (Additional file 1: Figure S3). Assessment using a mouse specific TDP-43 antibody also demonstrated that despite reduced levels of endogenous murine TDP-43, (Additional file 1: Figure S2), there was evidence of both cytoplasmic and nuclear aggregation of endogenous TDP-43 in 8 week old TDP-43^WTxQ331K^ mice (Fig. 3b).

Ubiquitin (Fig. 3c, Additional file 1: Figure S4) and p62 (Fig. 3d, Additional file 1: Figure S4) immunohistochemistry also revealed multiple small globular inclusions present in the neuronal cytoplasm and neuropil of TDP-43^WTxQ331K^ mouse ventral horns. Interestingly, there were a small number of p62 (sequestosome 1) positive inclusions in the spinal cord of 8 week old hTDP^Q331K^ animals (Additional file 1: Figure S4). Consistent with this, 24 month old TDP-43^Q331K^ mice demonstrated numerous small cytoplasmic p62 inclusions (Additional file 1: Figure S4), accompanied by occasional small TDP-43 inclusions in the neuropil (Additional file 1: Figure S3), although there was no evidence of any changes in ubiquitin staining in these animals. Co-labelling of myc-tagged TDP-43 with both ubiquitin and p62 demonstrated the presence of both proteins in TDP-43 inclusions (Figs. 3e-f) in TDP-43^WTxQ331K^ mice. Assessment of myc and ubiquitin positive aggregate containing cells in TDP-43^WTxQ331K^ animals (50–100 cells per mouse, n = 4; Fig. 3f) demonstrated that ~65 % of these cells displayed minimal nuclear myc-TDP staining, providing evidence of nuclear clearing in aggregate containing cells, which is supported by total and endogenous TDP-43 staining showing a loss of nuclear TDP-43 in a number of aggregate containing cells (Figs. 3a–b). In addition, co-staining studies showed the existence of many small p62 inclusions and a few small ubiquitin inclusions that did not colocalise with myc-TDP (Figs. 3e–f). Studies in other TDP-43 transgenic mouse models [34, 46] have shown evidence of eosinophilic aggregates consistent with mitochondrial aggregation, however, haemotoxylin and eosin staining did not reveal any overt signs of eosinophilic aggregates in our mice, regardless of genotype (Additional file 1: Figure S5) hence abnormal accumulation of mitochondria is unlikely to be a major pathological feature of these animals.

**Fig. 4:**
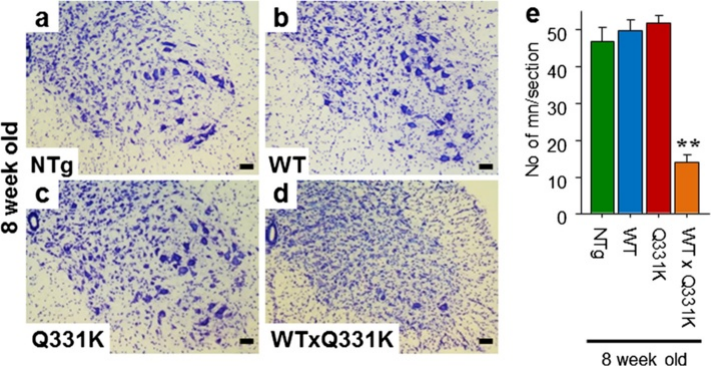
Co-expression of TDP-43^WT^ and TDP-43^Q331K^ in mice results in a loss of motor neurons from the anterior horn of the lumbar spinal cord. **a–d** Cresyl violet staining of motor neurons in the lumbar anterior horn of young NTg (**a**), TDP-43^WT^ (**b**), TDP-43^Q331K^ (**c**) and TDP-43^WTxQ331K^ (**d**) mice, showing a dearth of neurons present only in TDP-43^WTxQ331K^ animals (scale bar: 50 μm). **e** Cell counting of motor neurons in the lumbar anterior horn showed a significant loss of approximately 70 % of motor neurons in TDP-43^WTxQ331K^ mice compared with age matched non-transgenic and single transgenic animals. (** *p* < 0.001 vs all other age-matched genotypes)

Neuronal cell counting demonstrated a significant (~70 %) loss of α-motor neurons in the anterior horn of the lumbar spinal cord in TDP-43^WTxQ331K^ animals compared to their age-matched TDP-43^WT^ (*p* < 0.001), TDP-43^Q331K^ (*p* < 0.001) and non-transgenic (*p* < 0.001) littermates (Fig. 4). In the anterior horn, this was associated with a robust increase in astrogliosis, accompanied by mild microgliosis (Additional file 1: Figure S6) A moderate level of microgliosis was also found in the white matter tracts, and there was evidence of mild increases in astrogliosis in the anterior horn of single transgenic age-matched animals (Additional file 1: Figure S6). In 24 month old animals, a non-significant 25 % loss (*p* = 0.074) of α-motor neurons in the anterior horn (Additional file 1: Figure S7) was accompanied by a robust increase in astrogliosis in the TDP-43^Q331K^ transgenic animals compared to their non-transgenic and TDP-43^WT^ aged –matched littermates. These changes were accompanied by a modest microglial increase in the white matter tracts (Additional file 1: Figure S6), In addition, assessment of large caliber α-motor axons (>3.5 μm diameter) in the L5 nerve root demonstrated a significant (~46 %) loss in TDP-43^WTxQ331K^ mice at 8 weeks (Additional file 1: Figure S8), with no significant change observed in single transgenic animals at either age. Interestingly, there was a decline in the number of motor axons in all genotypes at 24 months of age, which is consistent with previous reports suggesting that C57Bl/6 mice display a subclinical loss of myelin during aging [47]. A trend to a further reduction (~12 %; *p* = 0.25; Additional file 1: Figure S8) in numbers of motor axons was seen in the phenotypic 24 month old TDP-43^Q331K^ animals. The loss of myelin reported in C57Bl/6 mice may partially mask the reduction in α-motor axons in aged TDP-43^Q331K^ animals. The loss of α-motor axons in TDP-43^WTxQ331K^ animals is accompanied by degenerating axons, characterised by the appearance of vacuolization and myelin defects (Additional file 1: Figure S8).

Muscle histology from end-stage TDP-43^WTxQ331K^ mice showed group atrophy of muscle fibres (Additional file 1: Figure S8) characteristic of denervation seen in muscle from ALS patients, while age-matched single transgenic animals were not significantly different from their non-transgenic littermates. Analysis of aged TDP-43^Q331K^ animals revealed a mild disorganisation of muscle fibres compared to age-matched littermates (Additional file 1: Figure S8) and immunoflourescent analysis revealed a significant reduction in the number of intact neuromuscular junctions (NMJ) in TDP-43^WTxQ331K^ mice (~60 %; *p* < 0.001; Additional file 1: Figure S8) coupled with significant structural disruption, indicated by a reduction in NMJ area (Table S1) compared to their non-transgenic and TDP-43^WT^ littermates. There was also a mild, reduction in NMJs in age-matched TDP-43^Q331K^ animals (~15 %; *p* = 0.06; Additional file 1: Figure S8). Consistent with data from young animals, 24 month old TDP-43^Q331K^ animals displayed a significant reduction in the number of intact NMJs (~35 %; *p* < 0.05; Additional file 1: Figure S8), accompanied by a reduction in area (Table S1), demonstrating an age-dependent degeneration of the NMJs in the TDP-43^Q331K^ animals.

### Brain pathology in TDP-43^Q331K^ mice is potentiated by co-expression with TDP-43^WT^ without loss of nuclear TDP-43

**Fig. 5:**
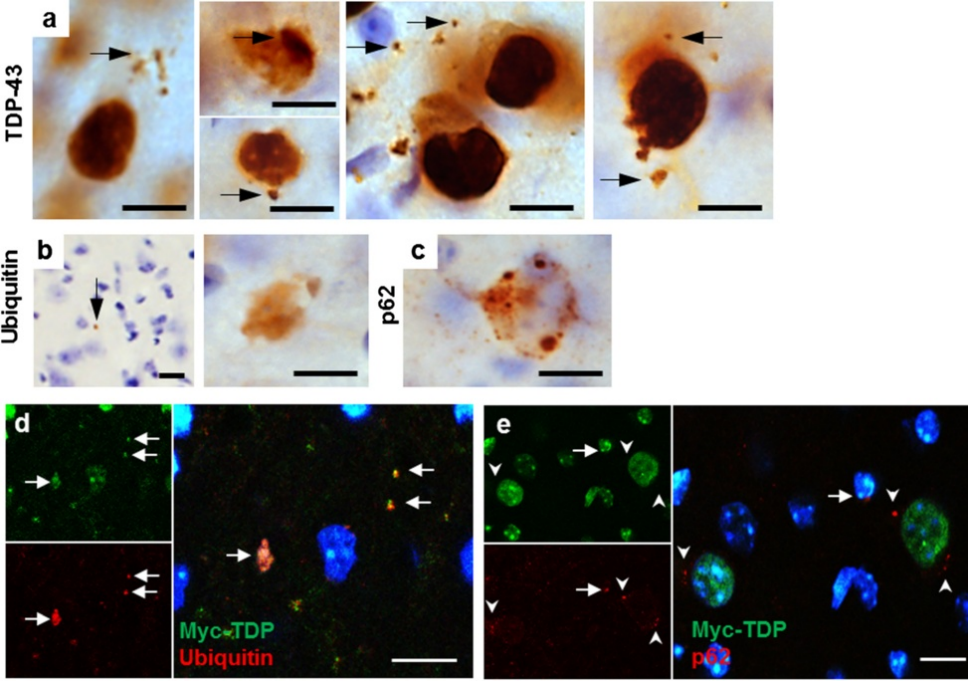
Co-expression of TDP-43^WT^ and TDP-43^Q331K^ in mice results in ubiquitin and p62 positive TDP-43 inclusions in the layer V region of the motor and somatosensory cortex. **a** Small TDP-43 inclusions were present in large pyramidal cells from the layer 5 region of the motor and somatosensory cortices of TDP-43^WTxQ331K^ mice (arrows), accompanied by ubiquitin (**b**) and p62 (**c**) aggregates (scale bar: 10 μm). Fluorescent co-labelling of myc-TDP and ubiquitin (**d**) or p62 (**e**) in the motor cortex of TDP-43^WTxQ331K^ animals shows aggregates that are positive for both myc-TDP and p62/ubiquitin (arrows), together with myc-TDP negative, p62 inclusions (arrowheads). (scale bar : 10 μm)

TDP-43 positive puncta without loss of nuclear TDP-43 were identified in layer V of the motor and somatosensory cortices in TDP-43^WTxQ331K^ mice (Fig. 5a, Additional file 1: Figure S9), which were not apparent in age-matched non-transgenic, TDP-43^WT^ or TDP-43^Q331K^ single transgenic littermates (Additional file 1: Figure S9). Ubiquitin and p62 staining in the TDP-43^WTxQ331K^ animals shows similar small inclusions together with occasional larger inclusions in the same region (Figs. 5b–c, Additional file 1: Figure S9), accompanied by an increase in diffuse ubiquitin staining in the pyramidal cells compared to age matched single and non-transgenic littermates (Additional file 1: Figure S9). As seen in the cord, cortices from 8 week old TDP-43^Q331K^ animals contain occasional small cytoplasmic p62 inclusions, together with occasional small ubiquitin inclusions while phenotypic 24 month old TDP-43^Q331K^ animals also display evidence of small ubiquitin and p62 inclusions (Additional file 1: Figure S10) that are not apparent in age-matched non-transgenic or TDP-43^WT^ animals. There is also evidence of small TDP-43 inclusions in the aged TDP-43^Q331K^ mice (Additional file 1: Figure S9) that are not apparent in aged non-transgenic or TDP-43^WT^ mice. Co-labelling of myc-TDP and ubiquitin (Fig. 5d) demonstrates a clear colocalisation of these proteins in large inclusions identified in the cortex of TDP-43^WTxQ331K^ mice, together with partial colocalisation in smaller puncta. In contrast, although there is evidence of occasional perinuclear TDP-43 and p62 colocalisation, multiple small cytoplasmic p62 inclusions throughout the cortex show no evidence of myc-TDP colocalisation (Fig. 5e), again suggesting that this p62 may be targeting some other, as yet unidentified protein or proteins.

**Fig. 6:**
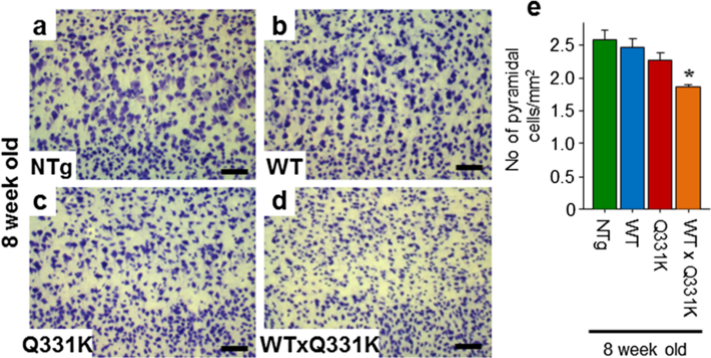
Co-expression of TDP-43^WT^ and TDP-43^Q331K^ in mice results in a loss of pyramidal neurons from the layer V region of the motor and somatosensory cortex. (a-d) Cresyl violet staining of cells in the layer V region of the cortex of young NTg (**a**), TDP-43^WT^ (**b**), TDP-43^Q331K^ (**c**) and TDP-43^WTxQ331K^ (**d**) mice, showing a dearth of large pyramidal neurons in TDP-43^WTxQ331K^ animals, together with a mild reduction in TDP-43^Q331K^ mice. **e** Cell counting of large pyramidal cells in the cortex showed a significant loss of approximately 28 % of large pyramidal neurons in TDP-43^WTxQ331K^ mice compared with age matched non-transgenic and TDP-43^WT^ animals. Young TDP-43^Q331K^ single transgenic animals showed a mild, non-significant loss of approximately 12 % of pyramidal cells. (* *p* < 0.05 vs age-matched NTg and TDP-43^WT^ animals)

Pyramidal neuron counts in layer V reveal a significant reduction in number in the cortex of TDP-43^WTxQ331K^ mice (~28 %; *p* = 0.007) compared to their single and non-transgenic littermates (Fig. 6). This loss is accompanied by a profound increase in GFAP immunoreactive astrogliosis specific to layer V in TDP-43^WSTxQ331K^ mice (Additional file 1: Figure S11). Interestingly, young TDP-43^Q331K^ single transgenic animals also show a mild increase in astrogliosis in this region (Additional file 1: Figure S11), despite the lack of motor phenotype at this age. There is also a trend towards a reduction in pyramidal neurons in the cortex of these animals (~12 %), but this does not reach significance (*p* = 0.253). In contrast, young TDP-43^WT^ single transgenic animals do not differ from their non-transgenic littermates (Fig. 6). The increase in astrogliosis in TDP-43^WTxQ331K^ mice is accompanied by a mild increase in CD68 positive microglioisis in the same region (Additional file 1: Figure S11), demonstrating a significant pathological impact of coexpression of the WT and mutant transgenes on the cortex, specifically the motor cortex and upper motor neurons, in TDP-43^WTxQ331K^ animals. Consistent with the data from young animals, 24 months old TDP-43^Q331K^ single transgenic animals show increased astro- and microgliosis in the layer V of the cortex compared to their non-transgenic and TDP-43^WT^ littermates (Additional file 1: Figure S11). These increases in the cortical neuroinflammatory response are accompanied by a loss of pyramidal neurons from the cortex (~14 %, *p* = 0.035; Additional file 1: Figure S12).

### Motor dysfunction and death in WTxQ331K mice is accompanied by a significant increase in pathogenic TDP-43

**Fig. 7:**
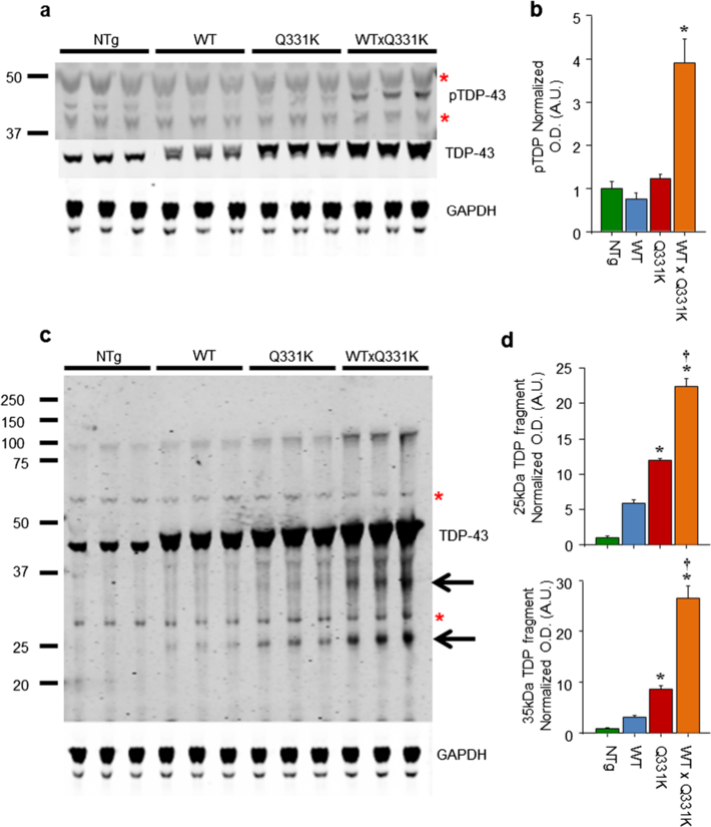
Co-expression of TDP-43^WT^ and TDP-43^Q331K^ in mice results increases levels of pathological phosphorylated TDP-43 and C-terminal fragments. **a** Western blotting from brains demonstrated an increase in phosphorylated TDP-43 (pTDP) in TDP-43^WTxQ331K^ mice, compared to their single and non-transgenic littermates (asterisks indicate non-specific bands). **b** Quantification of pTDP showing a significant increase only in the TDP-43^WTxQ331K^ animals (* *p* < 0.05 vs. all other genotypes). **c** Western blotting of total TDP-43 showing an increase in the levels of both 25 kDa and to a lesser extent 35 kDa TDP-43 fragments (arrows) in transgenic mice compared to their non-transgenic littermates (asterisks indicate non-specific bands). **d** Quantification of the 25 kDa and 35 kDa fragments, demonstrating an expression and mutation dependent increase in both fragments that reaches significance in TDP-43^Q331K^ and TDP-43^WTxQ331K^ animals (* *p* < 0.05 vs. NTg animals; †*p* < 0.05 vs. TDP-43^WT^ animals)

Immunoblot analysis of phosphorylated TDP-43 (pTDP) reveals a significant increase in levels in end-stage TDP-43^WTxQ331K^ animals compared to age-matched single and non-transgenic littermates (Figs. 7a-b). This is accompanied by an increase in the levels of both 25 and 35 kDa fragments of TDP-43 (Figs. 7c-d) providing further evidence of a pathological profile similar to that seen in human patients in these compound transgenic mice. Lesser, but still significant increases in both TDP-43 fragments are also present in 8 week old TDP-43^Q331K^ animals in the absence of significant increases in pTDP.

**Fig. 8:**
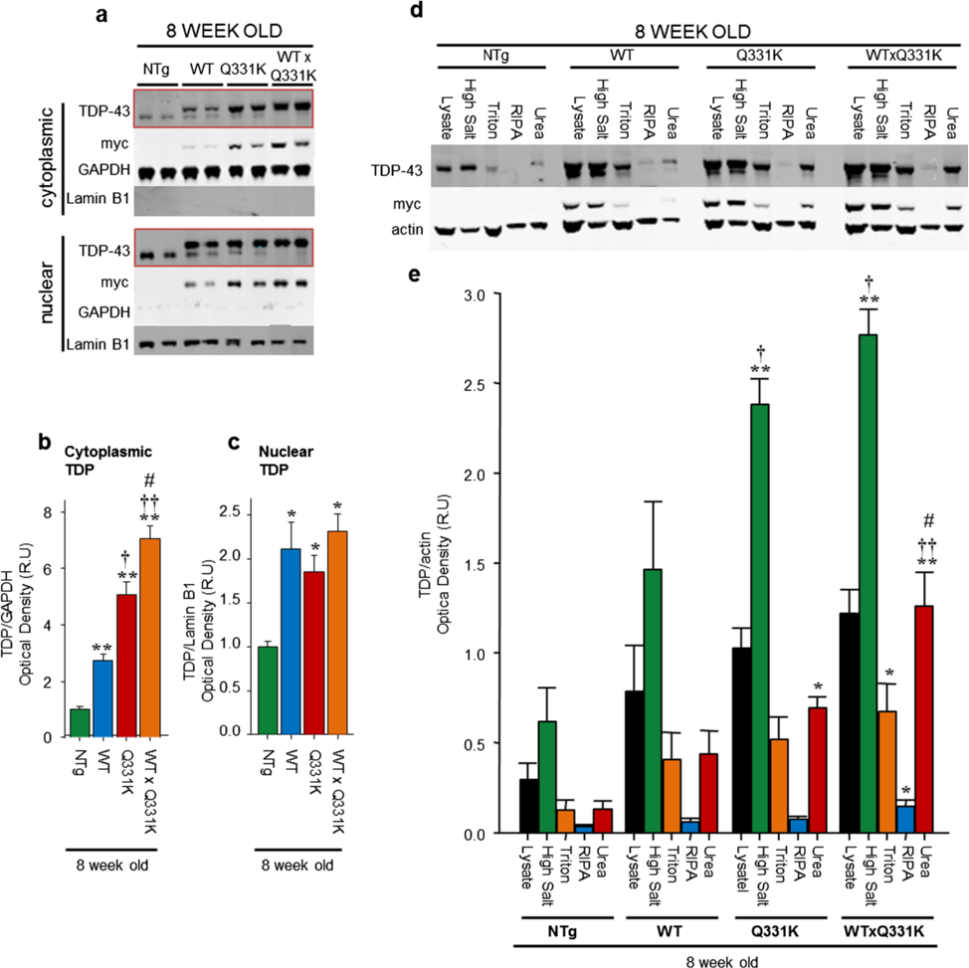
Co-expression of TDP-43^WT^ and TDP-43^Q331K^ in mice results in an increase in cytoplasmic and insoluble TDP-43. **a** Western blotting of nuclear and cytosolic fractions from brains demonstrated an increase in cytosolic and nuclear TDP-43 levels in young transgenic animals compared to their non-transgenic littermates. GAPDH was used as the marker for the cytosolic fraction, and Lamin B1 for the nuclear fraction. **b** Quantification of cytosolic TDP-43 levels (using the bands indicated in the red boxes), showing an expression and mutation dependent increase in TDP-43 levels in all transgenic animals (** *p* < 0.001 vs. NTg; †*p* < 0.05; ††*p* < 0.001 vs. TDP-43^WT^, #*p* < 0.05 vs. TDP-43^Q331K^). **c** Quantification of nuclear TDP-43 levels (using the bands indicated in the red boxes), showing a similar increase in TDP-43 in all transgenic animals, regardless of mutation status (* *p* < 0.05 vs. NTg). **d** Western blotting of detergent fractionation of brain demonstrated an increase in soluble and insoluble TDP-43 in all transgenic animals. This increase was more pronounced in TDP-43^WTxQ331K^ and TDP-43^Q331K^ animals. **e** Quantification of detergent soluble and insoluble TDP-43 levels, showing a significant increase in TDP-43 in all fractions in TDP-43^WTxQ331K^ animals compared to NTg littermates, and in the insoluble urea fraction, compared to all other animals. Insoluble TDP-43 was also significantly increased in the urea fraction of TDP-43^Q331K^ single transgenic animals, as was soluble TDP-43 detected in the high salt fraction. (* *p* < 0.05; ** *p* < 0.001 vs. non-transgenic animals; †*p* < 0.05; †*p* < 0.001 vs. TDP-43^WT^ animals; #*p* < 0.05 vs. TDP-43^Q331K^ animals)

Assessment of TDP-43 cellular localisation reveals a significant increase in the level of both cytoplasmic and nuclear TDP-43 in all transgenic animals compared to age matched non-transgenic littermates (Figs. 8a-c, Additional file 1: Figure S12). TDP-43^WTxQ331K^ and TDP-43^Q331K^ animals both displayed a significant increase in cytoplasmic TDP-43 levels compared to TDP-43^WT^ mice indicating that the presence of the TDP-43^Q331K^ mutation enhances this mislocalisation. In contrast, there was no significant increase in nuclear TDP levels between the various transgenic animals (Fig. 8c, Additional file 1: Figure S13). This suggests that levels of nuclear TDP-43 are tightly regulated and could not account for neurodegenerative pathology.

Analysis of TDP-43 solubility demonstrates that although the majority of the protein is present in the high salt fraction, as previously reported [36], there is also a significant increase in the amount of TDP-43 identified in the urea fraction in the TDP-43^WTxQ331K^ mice (Figs. 8d–e), compared to their non-transgenic, TDP-43^WT^ and TDP-43^Q331K^ single transgenic littermates. This is consistent with the identification of TDP-43 positive inclusions in the brain and cord of these mice. These animals also display a general increase in TDP-43 insolubility, with increases also observed in the Triton and RIPA fractions. In addition, both young and aged TDP-43^Q331K^ single transgenic animals display an increase in detergent insoluble TDP-43 compared to age-matched non-transgenic animals (Figs. 8d–e, Additional file 1: Figure S13), hence there appears to be a correlation between levels of detergent insoluble TDP-43 and the degenerative pathological and behavioural motor phenotype observed in these mice.

## Discussion

TDP-43 is a predominantly nuclear protein but also shuttles to the cytoplasm fulfilling multiple roles in RNA processing [48, 49, 50]. Cytoplasmic TDP-43 inclusions are the pathological hallmark in ~90 % of ALS and 55 % of FTLD cases, [1, 2] and in many surviving neurons levels of nuclear TDP-43 are greatly reduced. Wild-type TDP-43 mis-accumulation can arise from many different gene defects which account for ~10 % of all cases. Multiple lines of evidence from cellular and animal studies indicate that the mis-accumulation of TDP-43 is directly toxic to neurons and glia but whether the mechanism is due to a loss of nuclear TDP-43 function or toxicity due to cytoplasmic aggregation, or a combination of the two, is unclear.

Here we describe the behavioural and pathological features of a line of transgenic mice expressing the ALS-linked mutant TDP-43^Q331K^. Single hemizygous TDP-43-43^Q331K^ animals developed a late-onset, age-dependent, progressive motor deficit detectable by reduced rotarod performance from 3 months of age, which slowly declines out to 24 months, but is not fatal. Aged TDP-43^Q331K^ mice have a mild loss of pyramidal neurons from cortical layer V and α-motor neurons from the lumbar spinal cord, often with TDP-43, ubiquitin and p62 inclusions in surviving cells. This is accompanied by a mild loss of motor axons and neuromuscular junctions peripherally and moderate micro- and astrogliosis in the cortex and spinal cord centrally. The presence of insoluble cytoplasmic TDP-43 inclusions without a reduction of nuclear TDP-43, implies that it is cytoplasmic TDP-43 aggregation that exerts a neurotoxic effect. Neurodegeneration in TDP-43^Q331K^ mice began much earlier as some layer V cortical neurons had ubiquitin and p62 inclusions with mild neuronal loss and astrocytosis even at 8 weeks. Histological changes in the spinal cord however were less marked, suggesting that TDP-43 toxicity may preferentially affect the motor cortex in these animals, progressing to motor neurons in the spinal cord as disease progresses.

Most surprisingly, while TDP-43^WT^ mice are indistinguishable from their non-transgenic littermates both behaviourally and pathologically, when crossed with the TDP-43^Q331K^ mutant, the compound hemizygotes produce an early-onset, rapidly progressive motor phenotype manifesting with tremor at 3–4 weeks, very poor rotarod performance and progressive hind limb paralysis, necessitating euthanasia by 8–10 weeks.. End-stage pathology revealed severe lower motor neuron loss from the lumbar spinal cord, accompanied by a loss of large calibre motor axons, and neuromuscular junctions with atrophic muscles. Many surviving spinal cord neurons contained TDP-43, ubiquitin and p62 positive cytoplasmic inclusions, and there was microglial and astrocytic activation in the anterior horn and throughout the white matter. In addition, increased levels of pTDP and 25 and 35 kDa fragments of TDP-43 were detected in these animals. Interestingly, brains from end-stage TDP-43^WTxQ331K^ mice also demonstrated key pathological features of patient tissues [1], including a moderate loss of pyramidal neurons in layer V of the cortex, accompanied by marked microglial and astrocytic activation, insoluble TDP-43, ubiquitin and p62 inclusions in surviving cortical neurons. Thus, these mice have many of the pathological hallmarks of ALS patient tissues [51, 1, 52].

Expression of TDP-43^WT^_,_ TDP-43^Q331K^ or TDP-43^WTxQ331K^ resulted in a ~40–65 % reduction in endogenous mouse TDP-43 levels as has been previously reported [36, 53] but this was not associated with neurodegeneration in WT mice, hence this reduction *per se* is unlikely to be responsible for the variable phenotypes and pathology seen. In all mutant and WT transgenic lines studied the levels of nuclear TDP-43 was increased ~1 times greater than non-transgenic littermates at 8 weeks and 0.5 times greater at 24 months (Fig. 8, Additional file 1: Figure S13). Thus, neither a decrease in endogenous mouse TDP-43 nor an increase in nuclear human TDP-43 is likely to be pathogenic. Neurodegeneration was only associated with an increase in cytoplasmic TDP-43 at either time point. Remarkably both early and late-onset mice recapitulate many of the pathological features of TDP-43 proteinopathy that typifies ALS providing a powerful model to explore disease mechanisms and conduct preclinical trials.

Evidence of “nuclear clearing” of TDP-43 from neurons containing cytoplasmic inclusions, similar to that seen in two-thirds of surviving aggregate containing cells in the TDP-43^WTxQ331K^ animals, has been reported in ALS and FTLD patients [4, 5] and in other mouse models [32, 38]. Correspondingly, while animals with one endogenous TDP-43 allele inactivated are generally healthy, but have mildly impaired grip strength [27] homozygous deletion of TDP-43 in mice is embryonic lethal [27, 28]. It is not surprising therefore that focused depletion of a vital gene such as TDP-43 results in a paralytic neurodegeneration [30]. Similarly, a reduction in nuclear TDP-43 in mouse forebrain causes neuronal loss and abnormal hind-limb clasping [53]. By comparing the pathological changes in TDP-43^WTxQ331K^ (cytoplasmic aggregation and nuclear clearing) and aged TDP-43^Q331K^ mice (only cytoplasmic aggregation) one might conclude that while the loss of nuclear TDP-43 may accelerate disease, it is not essential, whereas cytoplasmic aggregation of TDP-43 into insoluble inclusions in cortical and spinal neurons, as seen in aged TDP-43^Q331K^ and TDP-43^WTxQ331K^ mice, is sufficient to cause neurodegeneration, as has previously been reported in another line of mice expressing Q331K (2).

**Fig. 9:**
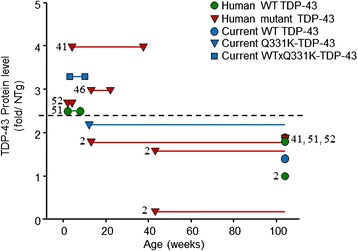
PrP driven overexpression of mutant or WT TDP-43 results in a dose-dependent impact on phenotype onset and survival. Data from all PrP-TDP-43 expression studies reported to date shows that overexpression of WT and/or mutant TDP-43 results in early phenotype onset and lethality when protein expression is approximately 2.3 fold or higher (dotted line) above endogenous. At expression levels lower than this, the mutant protein results in a non-lethal motor phenotype, while the WT protein does not induce a detectable phenotype. The data from our animals is consistent with this. For each mouse line, the first symbol represents onset, and the second represents survival. Circles represent WT lines, triangles represent mutant lines, and the squares represent the current compound transgenic line. Data from the current study is shown in blue, and numbers represent source reference

The simplest explanation for differences in the severity of phenotype and pathology is a dose-threshold effect. A comparison of protein levels, phenotype and survival in mice transgenic for human TDP-43 under PrP promoter (Fig. 9) shows that mice expressing human mutant TDP-43 appear to develop a phenotype at lower protein levels than those overexpressing the human WT as measured by western blot. This is consistent with previous reports suggesting that overexpression of disease mutant TDP-43 results in a more aggressive phenotype than human WT TDP-43 expressed at comparable levels [38]. Comparison of PrP driven overexpression shows that lethality is seen when TDP-43 is >2.3 fold for mutant or WT lines compared to non-transgenic endogenous mouse TDP-43. This is consistent with data from our lines (blue symbols in Fig. 9) where levels of TDP-43 in the brains of TDP-43^WT^ were 1.4 above endogenous in non-transgenic littermates, while TDP-43^Q331K^ mice were 2.2 fold and TDP-43^WTxQ331K^ 3.3 fold. Caution must be taken when interpreting these data however, as the methodology used to assess protein levels was not consistent between each study, hence a general dose effect, regardless of mutation status, cannot be ruled out. In the current study, we show that the addition of a ‘dose’ of human WT TDP-43 appears to trigger massive TDP-43 aggregation in TDP-43^Q331K^ mice and dramatically increases the toxicity such that 50 % of the mice are dead by 8 weeks. Assessment of the single transgenic animals used in this study have suggested that Q331K mutant TDP-43 has a greater tendency to aggregate in the cytoplasm than WT on its own, thus we hypothesise that Q331K aggregates provide a potent seed for the recruitment of human WT and endogenous mouse TDP-43 that results in nuclear clearing and rapid neurodegeneration. This hypothesis is supported by our finding that despite a general reduction in levels, endogenous mouse TDP-43 appears to be recruited to aggregates in the TDP-43^WTxQ331K^ mice. Although there is no overt evidence of endogenous TDP-43 aggregation in the TDP-43^Q331K^ single transgenic animals, the pathology seen in these mice is significantly reduced compared to the TDP-43^WTxQ331K^ animals, and this, combined with the reduction seen in endogenous TDP-43 is likely to make detection of any potential recruitment problematic.

## Conclusions

Our mice recapitulate many aspects of the clinical phenotype and pathological features of TDP-43-linked ALS with slow and rapidly progressive motor phenotypes. Although the occurrence of ALS-causing TDP-43 mutations is rare in the patient population, the close correlation between the pathology observed in these mice and that seen in the majority of TDP-ALS patients suggests that these animals provide a viable model for studying the impact and progression of TDP-43 pathology in the central nervous system, and help to elucidate mechanisms that may underlie disease progression. The aggregation of TDP-43 into insoluble cytoplasmic inclusions in cortical and spinal neurons was a feature of both aged TDP-43^Q331K^ and TDP-43^WTxQ331K^ mice while nuclear clearance was only seen in spinal cord motor neurons in TDP-43^WTxQ331K^ mice. Thus while nuclear clearance of TDP-43 may accelerate disease it does not appear to be essential to cause neurodegeneration and loss of nuclear TDP-43 function may not be a primary or disease-critical event. A more detailed exploration of disease mechanisms in slow and rapid disease models of TDP-43 proteinopathy will help elucidate novel drug targets and provide a more informative platform for preclinical trials.

## Additional files

Additional file 1:
**This file contains supplementary results as detailed in the manuscript.**
Additional file 2: Movie S1. This video shows a representative example of hind limb paralysis in a 6 week old TDP-43^WTxQ331K^ mouse, displaying a severely stilted gait, and an inability to lift their pelvis from the ground.
