# Supplementary material for: Wild type human TDP-43 potentiates ALS-linked mutant TDP-43 driven progressive motor and cortical neuron degeneration with pathological features of ALS
Source: Acta Neuropathol Commun. 2015 Jun 25;3:36. doi: 10.1186/s40478-015-0212-4 (PMC4479086; doi:10.1186/s40478-015-0212-4)
Supplement: Additional file 1: — This file contains supplementary results as detailed in the manuscript. [file 40478_2015_212_MOESM1_ESM.docx]

**Acta Neuropathoogica**

**Wild type human TDP-43 potentiates ALS-linked mutant TDP-43 driven progressive motor and cortical neuron degeneration with pathological features of ALS**

Jacqueline C Mitchell^1^, Remy Constable^1^, Eva So^1^, Caroline Vance^1^, Emma Scotter^1^, Leanne Glover^2^, Tibor Hortobagyi^1^, Eveline S. Arnold^3,*^, Shuo-Chien Ling^3^, Melissa McAlonis^3^, Sandrine Da Cruz^3^, Magda Polymenidou^3,#^, Lino Tessarolo^4^, Clotilde Lagier-Tourenne^3^, Don W Cleveland^3^, and Christopher E Shaw^1†^

^†^Corresponding author: [christopher.shaw@kcl.ac.uk](mailto:christopher.shaw@kcl.ac.uk) ^1^King’s Centre for Neurodegeneration Research, Kings College London, Department Of Basic and Clinical Neurosciences, Institute of Psychiatry, Psychiology and Neuroscience, London SE5 8AF

**Supporting Information**

| Genotype | 8 week NMJ area (µm^2^) | 24 month NMJ area (µm^2^) |
| --- | --- | --- |
| Non-transgenic | 485 ± 21.5 | 600 ± 26.7 |
| TDP-43^WT^ | 493 ± 20.4 | 615 ± 25.7 |
| TDP-43^Q331K^ | 469 ± 16.5 | 340 ± 18.2* |
| TDP-43^WTxQ331K^ | 265 ± 14* | N/A |

**Table S1** Co-expression of TDP-43^WT^ and TDP-43^Q331K^ in mice results in a significant reduction in NMJ area in 8 week old mice. Over expression of mutant TDP-43^Q331K^ also reduces NMJ area in 24 month old animals.
Data shown is mean ± SEM; *p<0.05 vs age matched single and non-transgenic animals


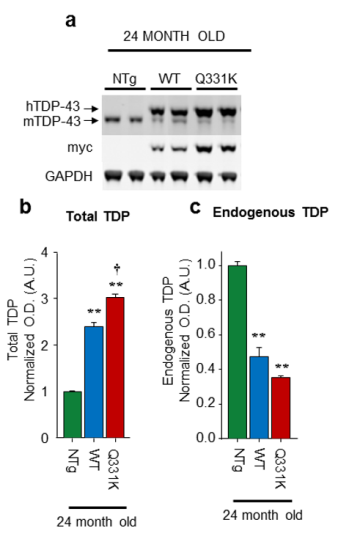


**Figure S1** Over expression of TDP-43^WT^ or TDP-43^Q331K^ decreases endogenous TDP-43 expression in 24 month old mice
(a) Western blotting of brain lysate from 24 month old NTg, TDP-43^WT^, and TDP-43^Q331K^ mice using an anti-TDP antibody showed a slight shift to a higher molecular weight due to the presence of the myc tag. There was an increase in total TDP43 expression in all transgenic animals, with higher expression in TDP-43^Q331K^ mice than TDP-43^WT^ animals (b).This increase was accompanied by a concomitant decrease in endogenous TDP43 expression (c)


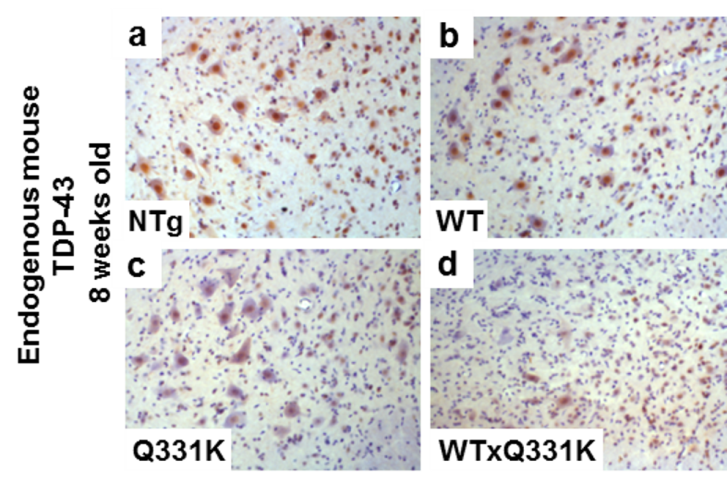


**Figure S2** Co-expression of TDP-43^WT^ and TDP-43^Q331K^ in mice results in a reduction in endogenous mouse TDP-43 levels
(a-d) Expression of endogenous mouse TDP-43 in the anterior horn of the lumbar spinal cord in NTg (a), TDP-43^WT^ (b), TDP-43^Q331K^ (c) and TDP-43^WTxQ331K^ (d) mice


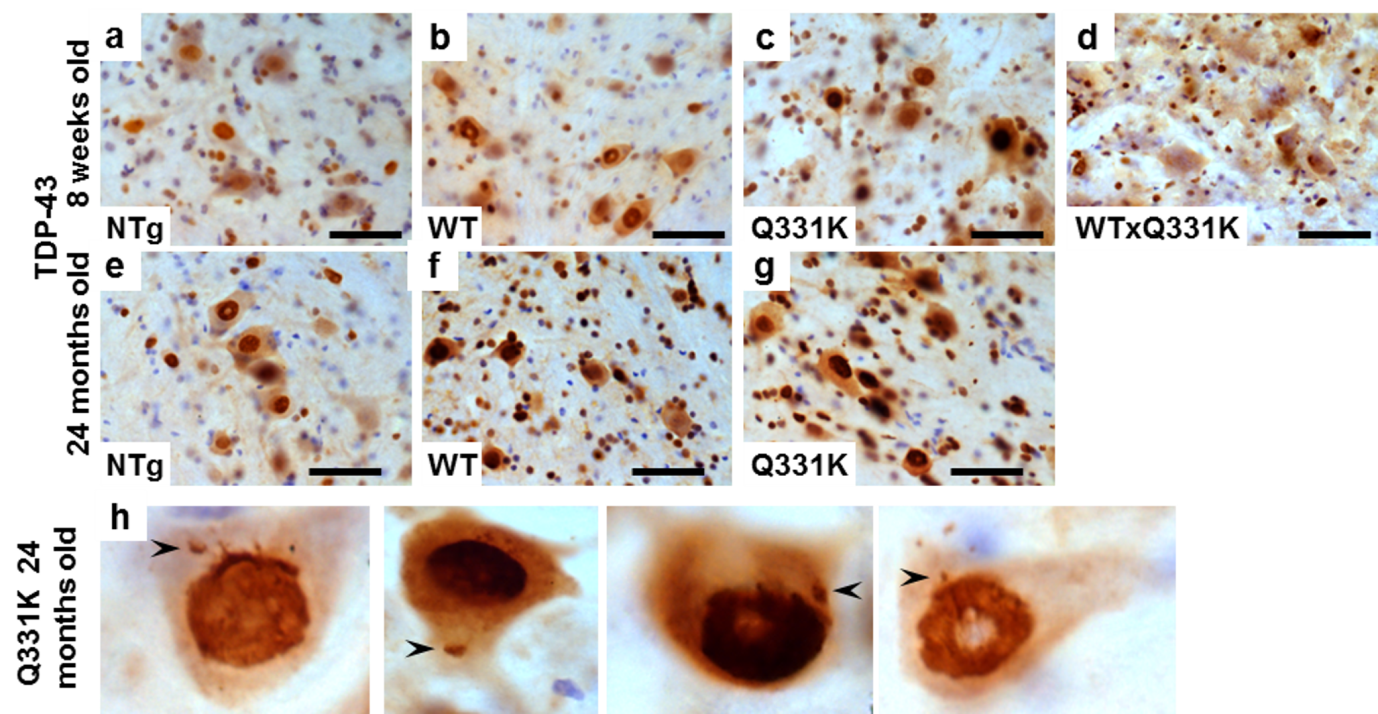


**Figure S3** Over expression of TDP-43^WT^ and/or TDP-43^Q331K^ in mice results in increased TDP-43 immunoreactivity in the anterior horn of the lumbar spinal cord
(a-g) TDP-43 expression in the lumbar spinal cord was increased in TDP-43^WT^ (b, f), TDP-43^Q331K^ (c, g) and TDP-43^WTxQ331K^ (d) animals compared to their age-matched NTg littermates (a, e) (scale bar: 50µm) (h) Small cytoplasmicTDP-43 inclusions were present in 24 month old TDP-43^Q331K^ animals (arrows), with no evidence of nuclear clearing of TDP-43.


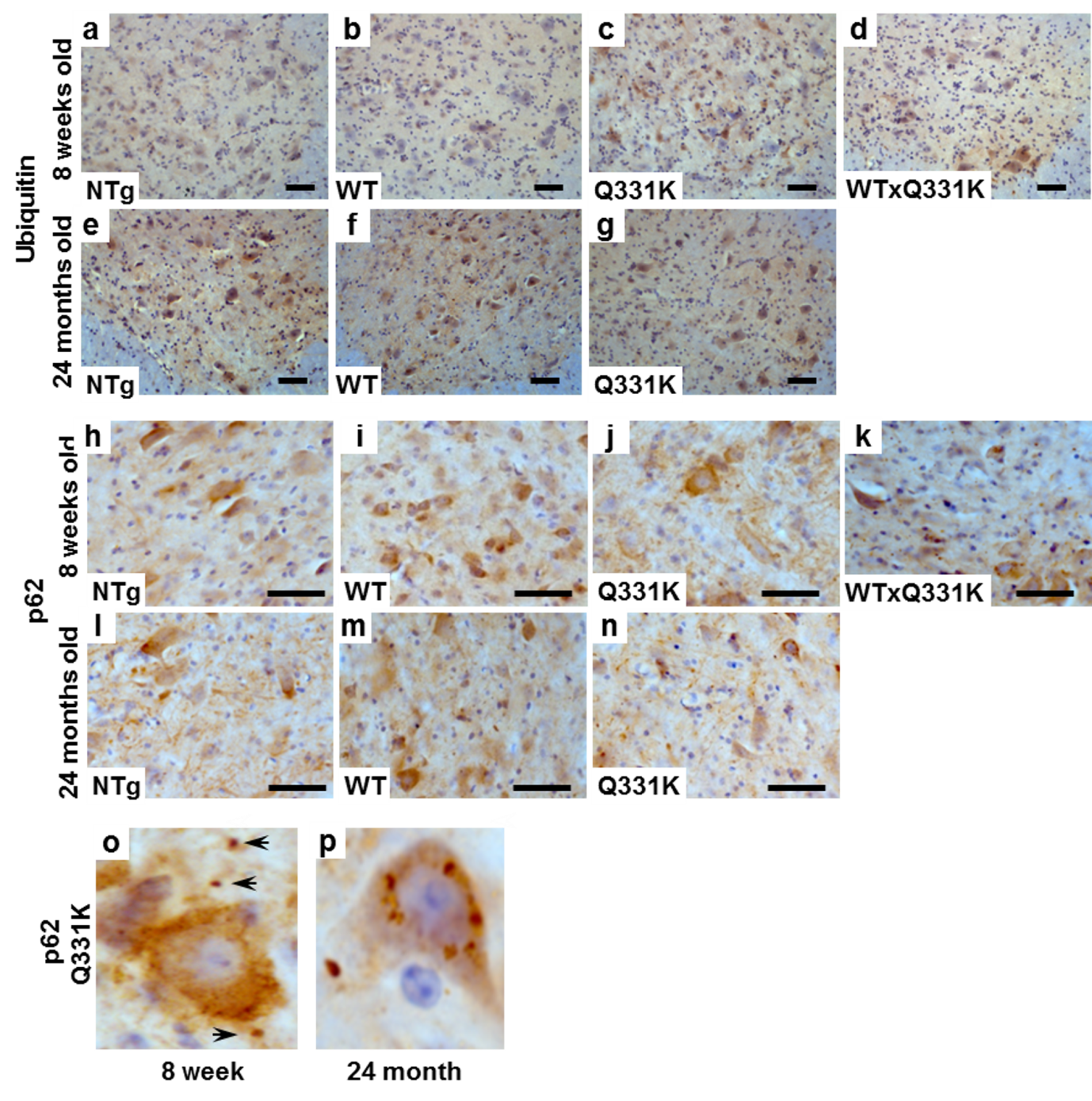


**Figure S4** Over expression of TDP-43^Q331K^ in mice results in p62 but not ubiquitin aggregation in the anterior horn of the lumbar spinal cord
(a-g) Ubiquitin expression in the lumbar spinal cord was not altered in TDP-43^WT^ (b, f) or TDP-43^Q331K^ (c, g) mice animals compared to their age-matched NTg littermates (a, e). In contrast, mild increases in ubiquitin were apparent in TDP-43^WTxQ331K^ animals (d). (h-n) Similarly, p62 levels were not altered in TDP-43^WT^ mice (I, m) compared to age-matched NTg littermates (h, l). In contrast, mild increases in p62 immunoreactivity were observed in both TDP-43^Q331K^ and TDP-43^WTxQ331K^ animals (scale bar: 50µm). (o-p) p62 aggregates were detectable occasionally in 8 weeks old (o), and more frequently in 24 month old (p) TDP-43^Q331K^ mice.


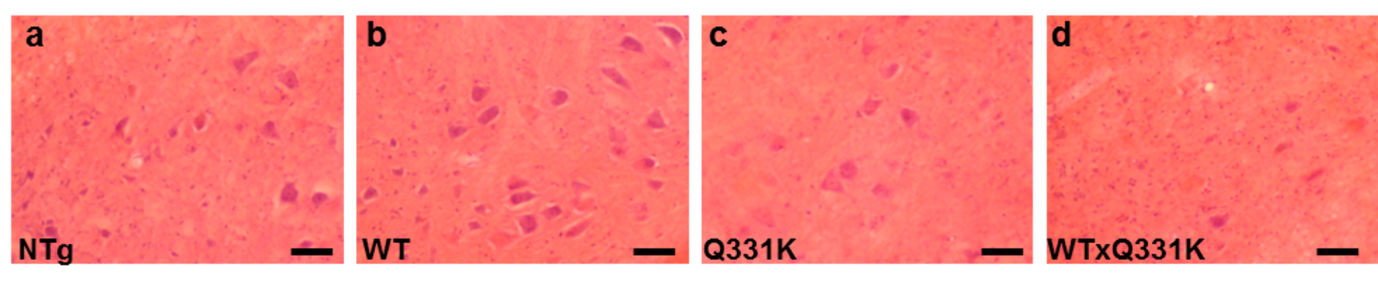


**Figure S5** Co-expression of TDP-43^WT^ and TDP-43^Q331K^ in mice does not result in perinuclear eosinophilic aggregates the anterior horn of the lumbar spinal cord.
(a-d) Haematoxylin and eosin staining of the lumbar spinal cord shows no evidence of eosinophilic aggregates in surviving motor neurons of TDP-43^WTxzQ331K^ mice (d) compared to their TDP-43^WT^ (b), TDP-43^Q331K^ (c), and NTg (a) littermates.


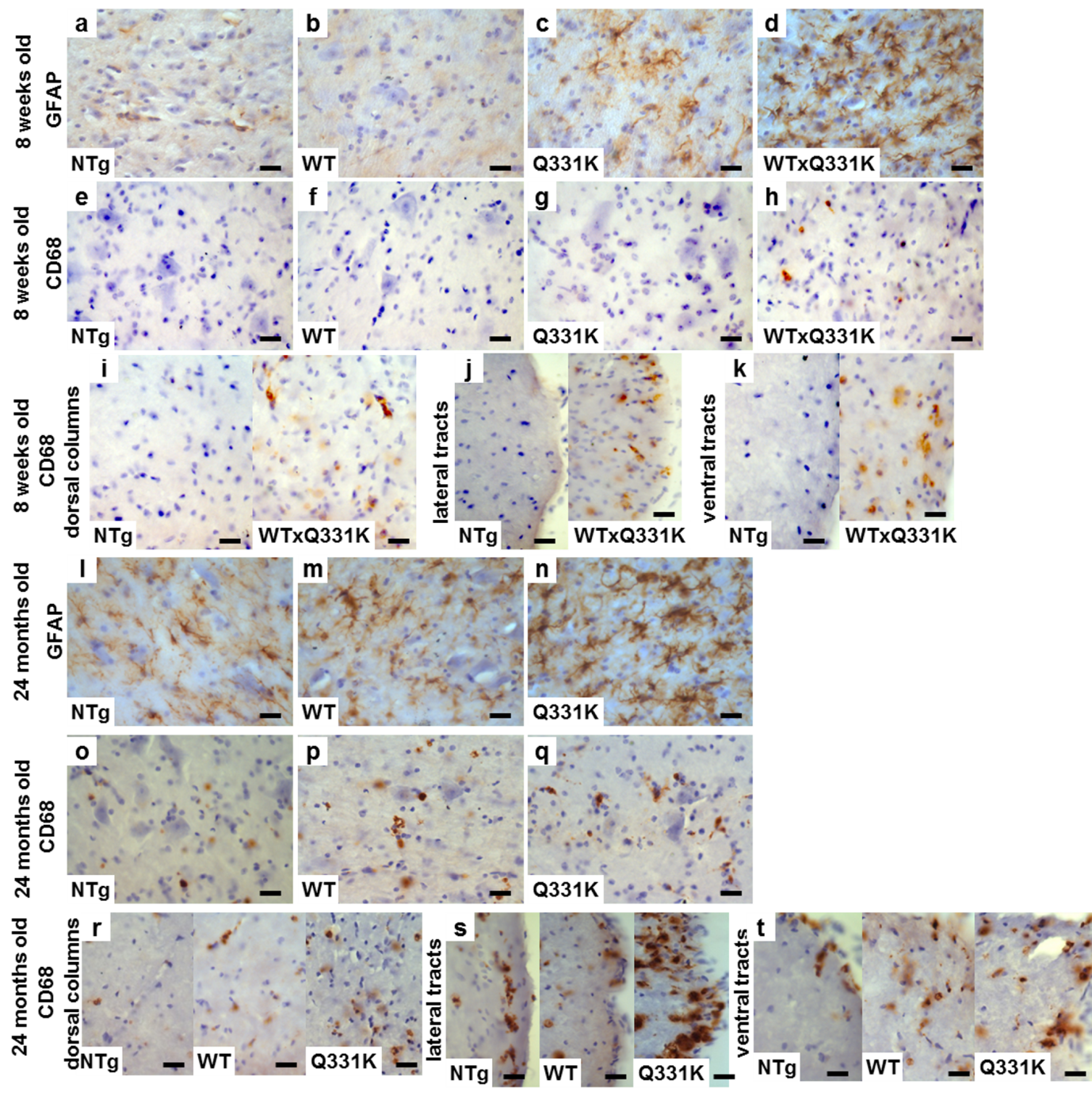


**Figure S6** Increased neuroinflammation in the cord of TDP-43^WTxQ331K^ compound transgenic and TDP-43^Q331K^ single transgenic animals.

(a-d) Significant increases in astrogliosis, detected using GFAP immunohistochemistry, were apparent in the anterior horn of the spinal cord in young TDP-43^WTxQ331K^ and TDP-43^Q331K^ animals (e-k) Mild increases in microglial activation, detected using CD68 immunohistochemistry, were apparent in the anterior horn of the spinal cord (e-h) and more dramatically in the dorsal columns (i), and lateral (j) and ventral (k) tracts of TDP-43^WTxQ331K^ mice. (l-n) Increases in astrogliosis were also present in the anterior horn of 24 month old TDP-43^Q331K^ mice compared to age-matched NTg and TDP-43^WT^ animals. (o-t) No major changes in microglial activation were observed in the anterior horn in any 24 month old animals, but significant increases were observed in the dorsal columns (r), and lateral (s) and ventral (t) tracts in TDP-43^Q331K^ mice compared to their NTg and TDP-43^WT^ littermates. (scale bar: 20µm)


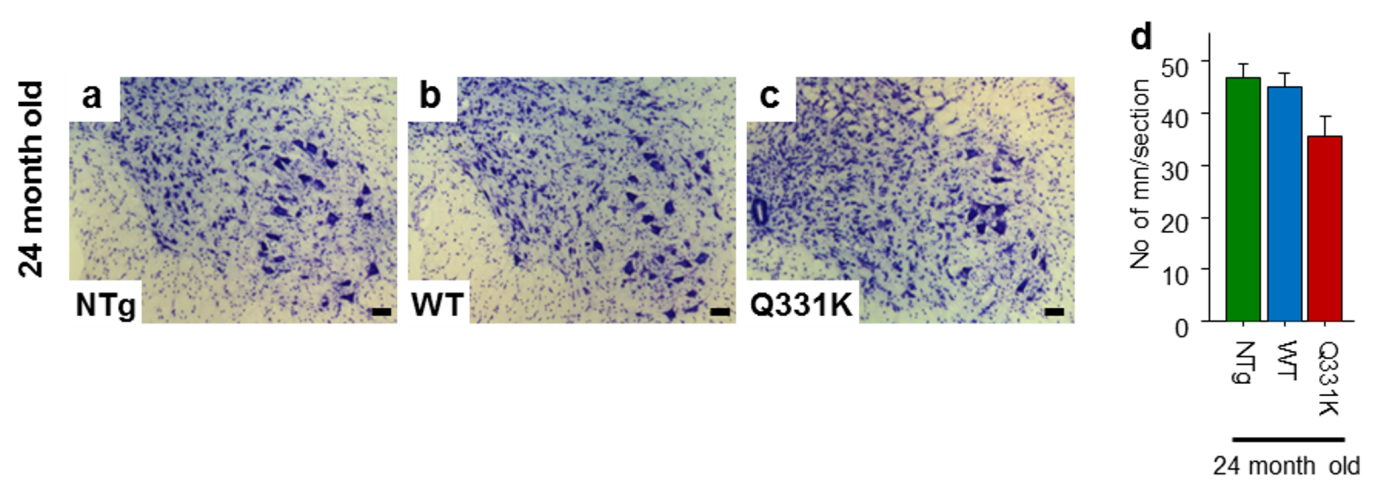


**Figure S7** Over expression of TDP-43^Q331K^ in mice results in a non-significant decrease in motor neurons in the lumbar spinal cord

(a-c) Cresyl violet staining of motor neurons in the lumbar anterior horn of aged NTg (a), TDP-43^WT^ (b) and TDP-43^Q331K^ (c) animals, showing a mild loss of neurons present only in the TDP-43^Q331K^ mice. (scale bar: 50µm) (d) Cell counting of motor neurons in the lumbar anterior horn showed a loss of approximately 25% of motor neurons compared to age matched NTg and TDP-43^WT^ mice, but this does not reach significance.).


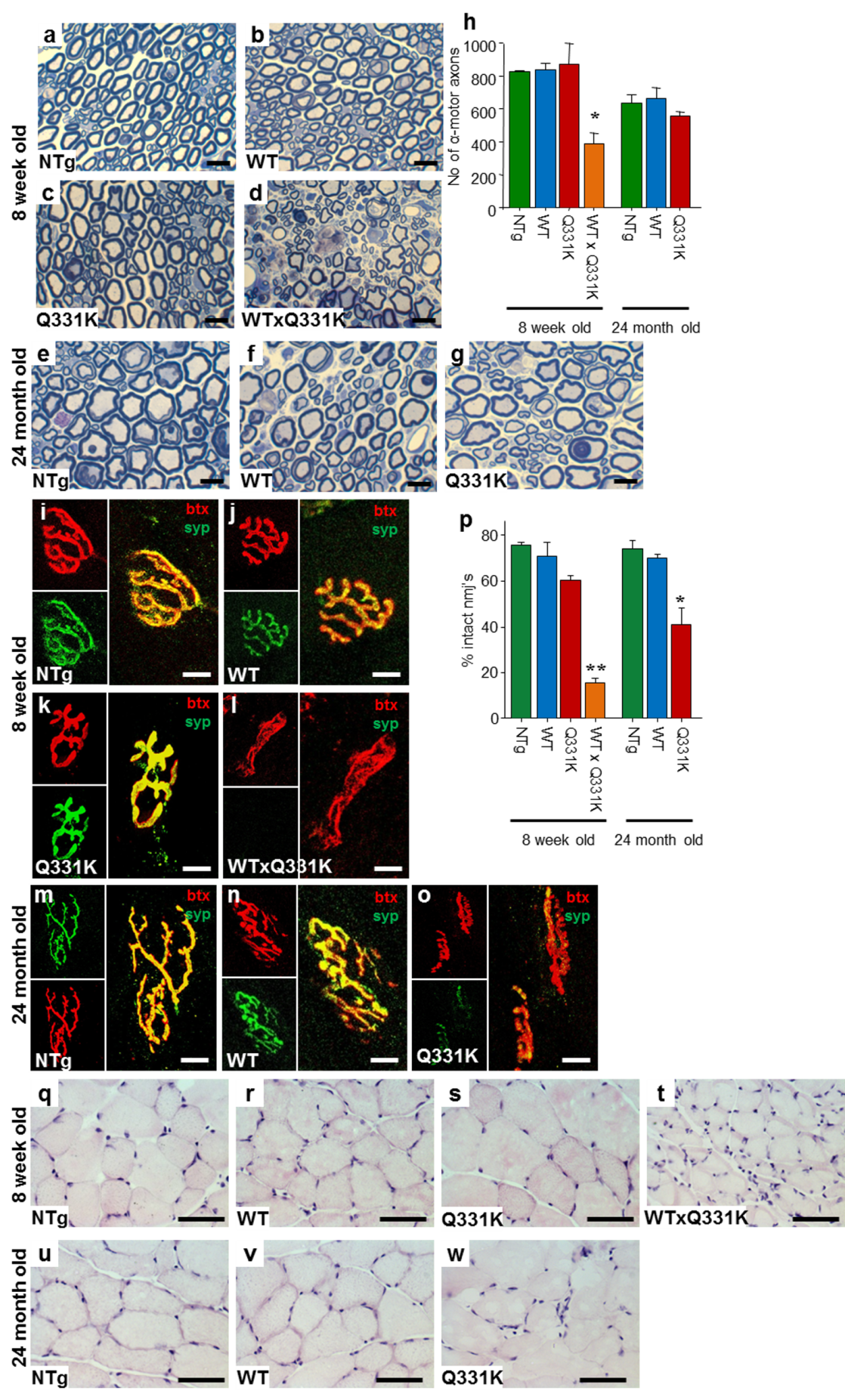


**Figure S8** Motor axon loss and muscle degeneration in TDP-43^WTxQ331K^ animals.

(a-d) Toluidine blue staining of L5 motor axon roots from young NTg (a), TDP-43^WT^ (b), TDP-43^Q331K^ (c) and TDP-43^WTxQ331K^ (d) mice, showing a reduction in the number of large motor axons, coupled with the appearance of vacuolization and myelin defects in TDP-43^WTxQ331K^ animals. (e-g) Toluidine blue staining of L5 motor axon roots from aged NTg (e), TDP-43^WT^ (f) and TDP-43^Q331K^ (g) mice, showing a mild, age related disruption in all animals, regardless of genotype. (scale bar:10µm) (h) Quantification of large (<3.5µm diameter) axons in L5 motor axon roots showed a significant loss of ~46% of motor axons in TDP-43^WTxQ331K^ mice compared to age matched non-transgenic and single transgenic animals. Aged animals display a reduction in the number of large motor axons regardless of genotype, with TDP-43^Q331K^ animals showing a mild, non-significant loss of approximately 12% compared to age-matched NTg and TDP-43^WT^ animals. (*****p<0.05 vs all other age-matched genotypes). (i-l) Bungarotoxin and synaptophysin co-labelling of neuromuscular junctions from young NTg (i), TDP-43^WT^ (j), TDP-43^Q331K^ (k) and TDP-43^WTxQ331K^ (l) mice, showing a loss of synaptohysin staining, coupled with a reduction in size and disorganization of the NMJ structure in TDP-43^WTxQ331K^ animals. (m-o) Bungarotoxin and synaptophysin co-labelling of neuromuscular junctions from aged NTg (m), TDP-43^WT^ (n) and TDP-43^Q331K^ (o) animals, showing a partial loss of synaptophysin staining, coupled with a mild reduction in size and disorganisation of the NMJ structure in TDP-43^Q331K^ mice (scale bar:10µm). (p) Quantification of intact NMJs (complete bungarotoxin and synaptophysin colocalisation) showing a significant reduction of ~60% in TDP-43^WTxQ331K^ mice compared to age matched NTg and TDP-43^WT^ animals. Young TDP-43^Q331K^ mice also display ~15% non-significant reduction in the number of intact NMJs. This reduction is increased in 24 month animals, with TDP-43^Q331K^ mice showing ~35% loss compared to age matched NTg and TDP-43^WT^ animals. (*****p<0.05; ******p<0.001 vs. all other age-matched genotypes). (q-w) Haematoxylin and eosin staining of muscle showed moderate scattered and grouped muscle fibre atrophy, characteristic of motor degeneration in TDP-43^WTxQ331K^ mice, with some evidence of mild muscle fibre disorganisation in aged TDP-43^Q331K^ animals (scale bar: 50µm).


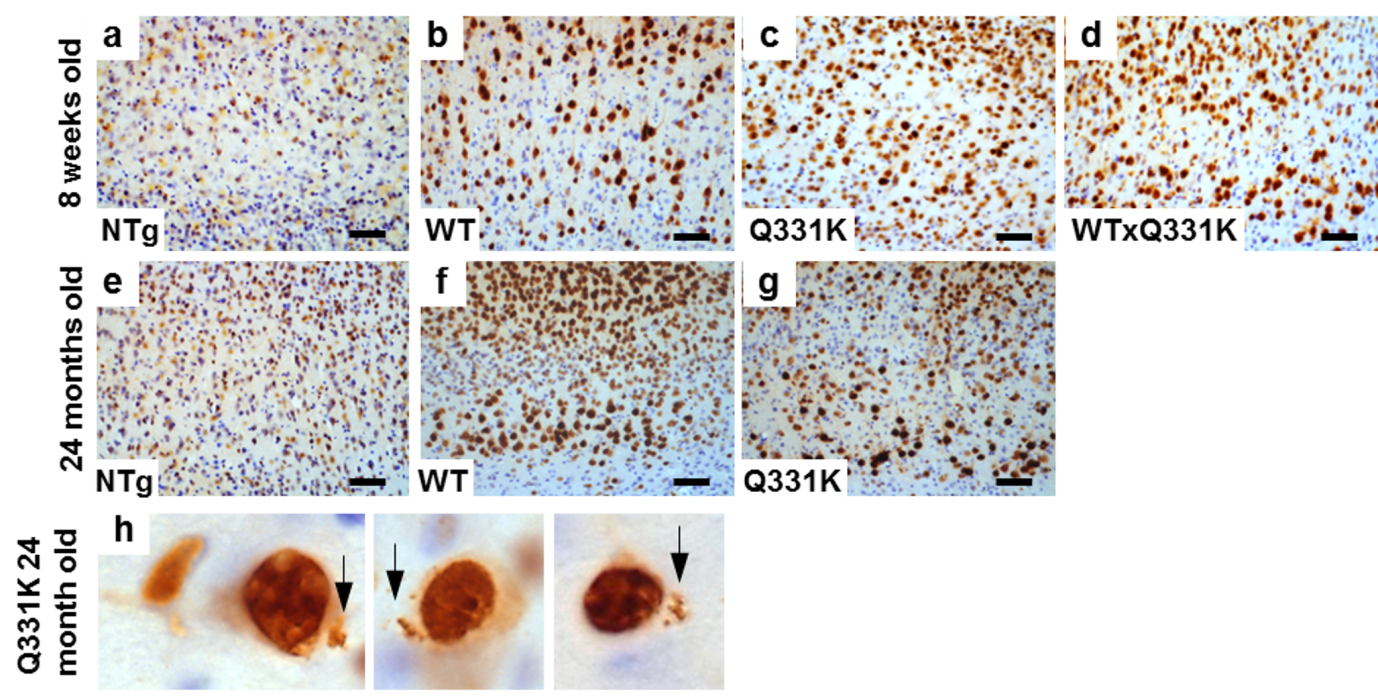


**Figure S9** Over expression of TDP-43^WT^ and/or TDP-43^Q331K^ in mice results in increased TDP-43 immunoreactivity in the motor and somatosensory cortices
(a-g) TDP-43 expression in the motor and somatosensory cortex was increased in TDP-43^WT^ (b, f), TDP-43^Q331K^ (c, g) and TDP-43^WTxQ331K^ (d) animals compared to their age-matched NTg littermates (a, e) (scale bar: 50µm) (h) Small cytoplasmicTDP-43 inclusions were present in 24 month old TDP-43^Q331K^ animals (arrows)


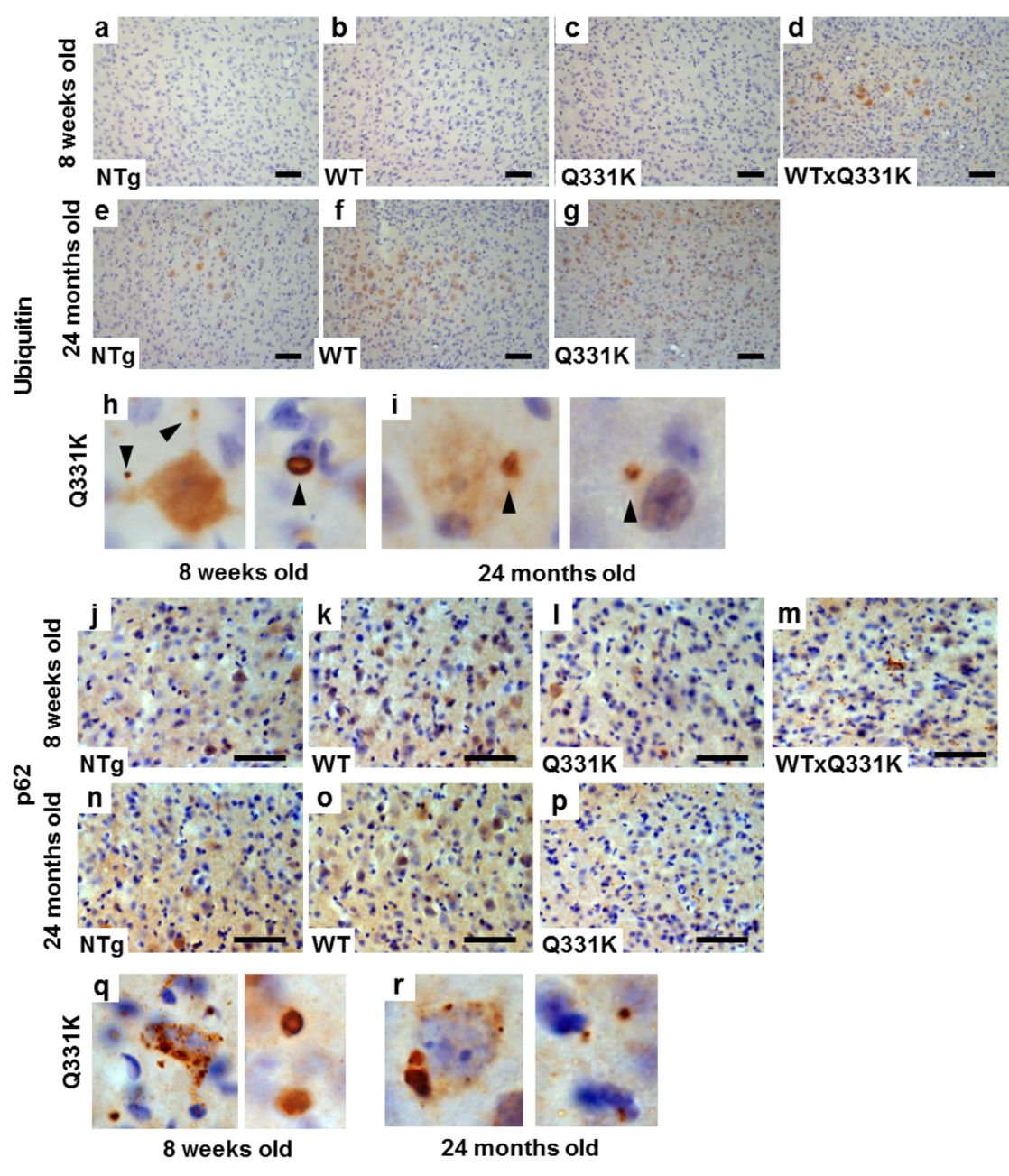


**Figure S10** Over expression of TDP-43^Q331K^ in mice results in p62 and ubiquitin aggregation in the motor cortex.
(a-g) Diffuse ubiquitin expression in the motor cortex was increased in TDP-43^WTxQ331K^ mice (d) compared to their age matched NTg (a) TDP-43^WT^ (b) or TDP-43^Q331K^ (c) littermates. Mild increases in diffuse ubiquitin staining were apparent in 24 month old mice, compared to young animals but TDP-43^Q331K^ (g), TDP-43^WT^ (f) were not distinguishable from their age-matched NTg littermates (e). (h-i) However, high power analysis reveals the presence of a small number of ubiquitin aggregates in both 8 week old (h) and 24 month old (i) TDP-43^Q331K^ animals. (j-p) Similarly, p62 levels were not altered in TDP-43^WT^ mice (k, o) compared to age-matched NTg littermates (j, n). In contrast, mild increases in p62 immunoreactivity were observed in both TDP-43^Q331K^ and TDP-43^WTxQ331K^ animals (scale bar: 50µm). (q-r) p62 aggregates were detectable occasionally in 8 weeks old (q), and more frequently in 24 month old (r) TDP-43^Q331K^ mice.


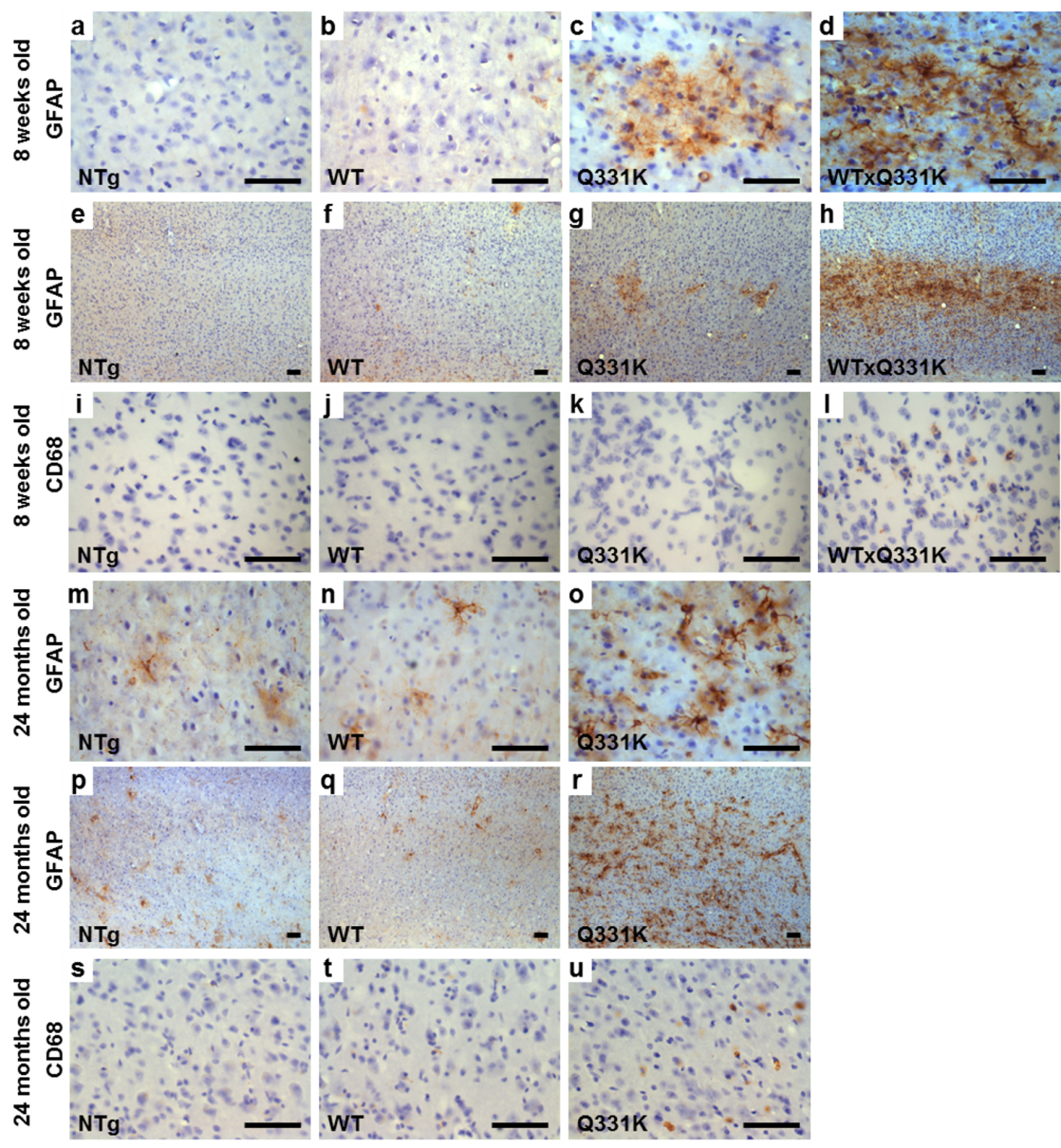


**Figure S11** Increased neuroinflammation in the layer V region of the cortex of TDP-43^WTxQ331K^ compound transgenic and TDP-43^Q331K^ single transgenic animals

(a-d) Significant increases in astrogliosis, detected using GFAP immunohistochemistry, were apparent in the layer V region of the cortex of young TDP-43^WTxQ331K^ and TDP-43^Q331K^ animals (e-h) Low power images of the cortex, demonstrating that the astrogliosis observed in the cortex of young TDP-43^WTxQ331K^ (h) and TDP-43^Q331K^ (g) animals is confined to the layer V region and is absent in NTg (e) and TDP-43^WT^ (f) mice. (i-l) Mild increases in microglial activation, detected using CD68 immunohistochemistry, were apparent in the layer V region of the cortex of TDP-43^WTxQ331K^ mice (l), but were not detectable in age-matched NTg (i), TDP-43^WT^ (j) or TDP-43^Q331K^ (k) animals. (m-o) Increases in astrogliosis were also present in the layer V region of the cortex 24 month old TDP-43^Q331K^ mice (o) compared to age-matched NTg (m) and TDP-43^WT^ (n) animals. (p-r) Low power images of the cortex of aged mice, demonstrating that the increases in astrogliosis observed in the cortex of TDP-43^Q331K^ animals (r), are again confined predominantly to the layer V region. (s-u) Mild increases in microglial activation were apparent in the layer V region of the cortex in 24 month old TDP-43^Q331K^ animals (u), which were not apparent in age-matched NTg (s) or TDP-43^WT^ (t) littermates. (scale bar: 50µm)


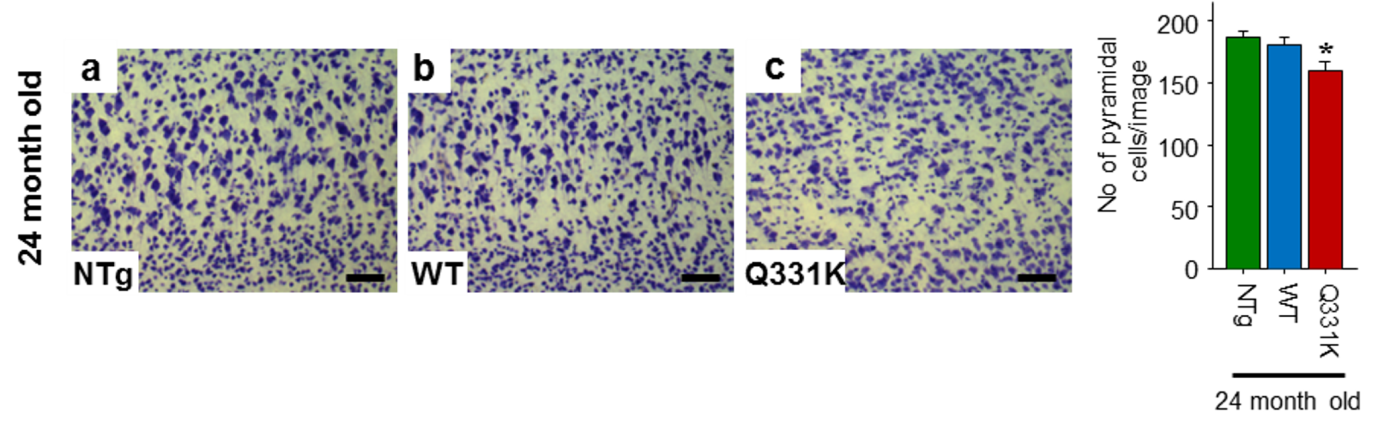


**Figure S12** Over expression of TDP-43^Q331K^ in mice results in a significant decrease in pyramidal neurons in the layer V region of the cortex

Cresyl violet staining of cells in the layer V region of the cortex of aged NTg (a), TDP-43^WT^ (b) and TDP-43^Q331K^ (c) mice, showing a mild reduction in the number of large pyramidal neurons in TDP-43^Q331K^ mice. (d) Cell counting of large pyramidal cells in the cortex showed a significant loss of approximately 14% of large pyramidal neurons in TDP-43^WTxQ331K^ mice compared with age matched non-transgenic and TDP-43^WT^ animals. (*****p<0.05 vs age-matched NTg and TDP-43^WT^ animals).


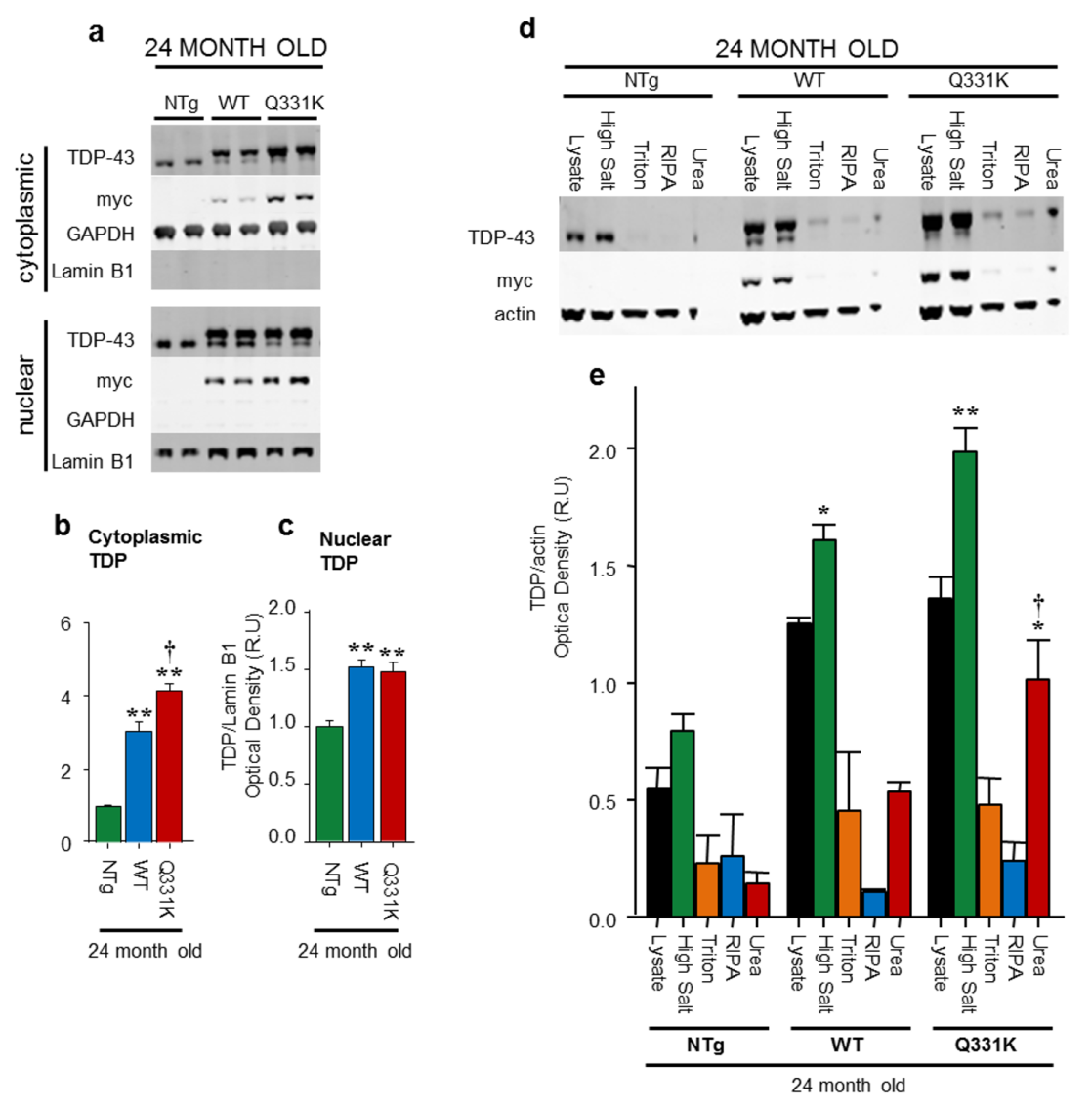


**Figure 13** Over expression of TDP-43^WT^ or TDP-43^Q331K^ in mice results in an increase in cytoplasmic and insoluble TDP-43 in 24 month old mice.

(a) Western blotting of nuclear and cytosolic fractions from brains demonstrated an increase in cytosolic and nuclear TDP-43 levels in 24 month old transgenic animals compared to their non-transgenic littermates. GAPDH was used as the marker for the cytosolic fraction, and Lamin B1 for the nuclear fraction. (b) Quantification of cytosolic TDP-43 levels, showing an expression and mutation dependent increase in TDP-43 levels in all transgenic animals, (******p<0.001 vs. NTg; †p<0.05 vs. TDP-43^WT^ ). (c) Quantification of nuclear TDP-43 levels showing a similar increase in TDP-43 in all transgenic animals, regardless of mutation status (******p<0.001 vs. NTg). (d) Western blotting of detergent fractionation of brain demonstrated an increase in soluble and insoluble TDP-43 in all transgenic animals. This increase was more pronounced in TDP-43^Q331K^ animals. (e) Quantification of detergent soluble and insoluble TDP-43 levels, showing a significant increase in soluble TDP-43 in the high-salt fraction of both TDP-43^WT^ and TDP-43^Q331K^ animals, which is significantly enhanced in the TDP-43^Q331K^ mice, coupled with a significant increase in insoluble TDP-43 in the urea fraction of TDP-43^Q331K^ single transgenic animals. (*****p<0.05; ******p<0.001 vs. non-transgenic animals; **†**p<0.05; **†**p<0.001 vs. TDP-43^WT^ animals; **#**p<0.05 vs. TDP-43^Q331K^ animals).
